# Supplementary material for: STAT3 sustains tumorigenicity following mutant KRAS ablation
Source: EMBO Rep. 2025 Aug 26;26(20):4900–22. doi: 10.1038/s44319-025-00563-w (PMC12549880; doi:10.1038/s44319-025-00563-w)
Supplement: Supplementary file 2 — Source data Fig. 1A to 1I [file 44319_2025_563_MOESM2_ESM.zip › Figure 1A-1I/Figure 1F/Figure 1F.pptx]

## Slide 1
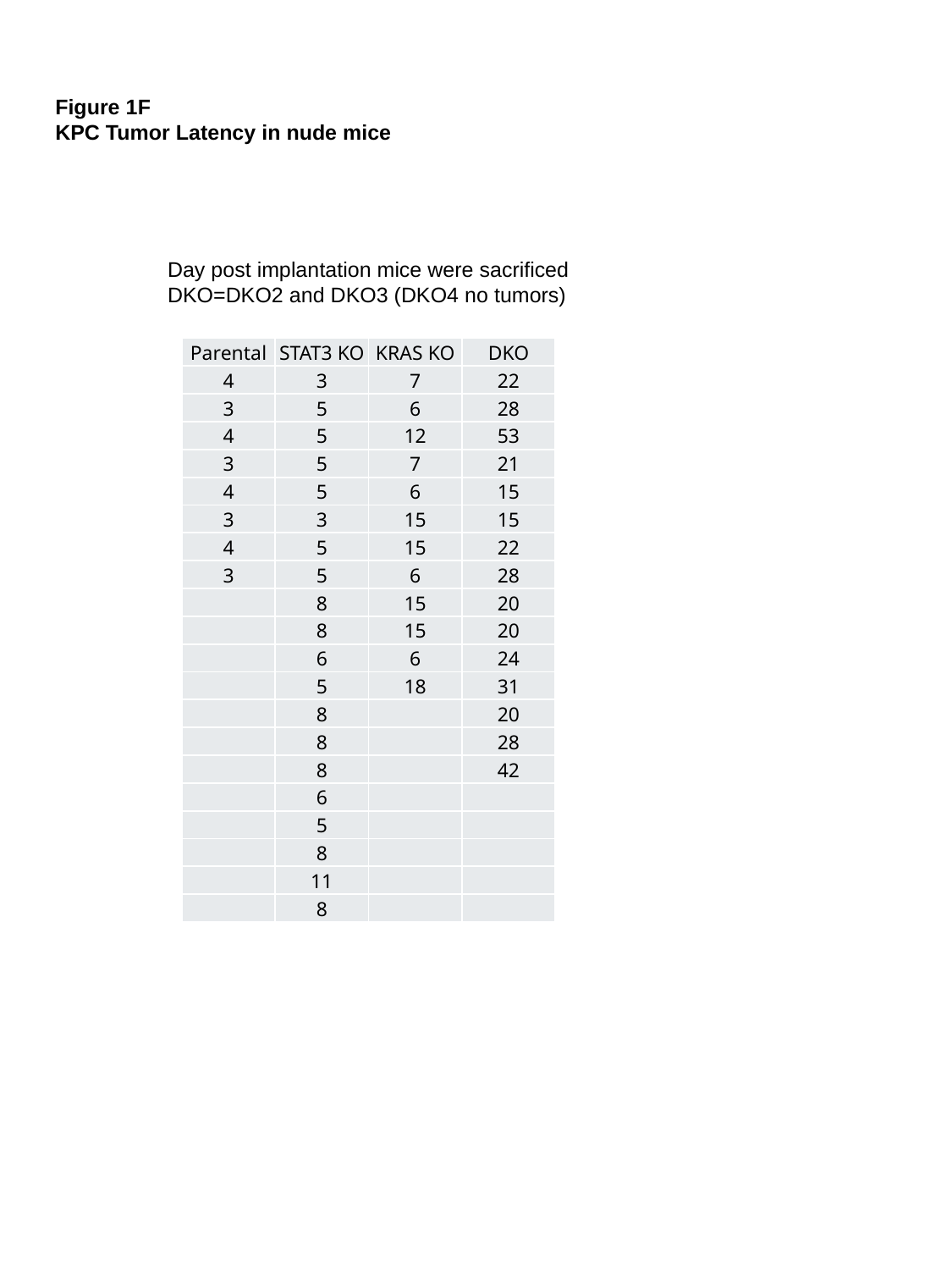

Figure 1F
KPC Tumor Latency in nude mice
Day post implantation mice were sacrificed
DKO=DKO2 and DKO3 (DKO4 no tumors)
| Parental | STAT3 KO | KRAS KO | DKO |
| --- | --- | --- | --- |
| 4 | 3 | 7 | 22 |
| 3 | 5 | 6 | 28 |
| 4 | 5 | 12 | 53 |
| 3 | 5 | 7 | 21 |
| 4 | 5 | 6 | 15 |
| 3 | 3 | 15 | 15 |
| 4 | 5 | 15 | 22 |
| 3 | 5 | 6 | 28 |
| | 8 | 15 | 20 |
| | 8 | 15 | 20 |
| | 6 | 6 | 24 |
| | 5 | 18 | 31 |
| | 8 | | 20 |
| | 8 | | 28 |
| | 8 | | 42 |
| | 6 | | |
| | 5 | | |
| | 8 | | |
| | 11 | | |
| | 8 | | |
