# Supplementary material for: STAT3 sustains tumorigenicity following mutant KRAS ablation
Source: EMBO Rep. 2025 Aug 26;26(20):4900–22. doi: 10.1038/s44319-025-00563-w (PMC12549880; doi:10.1038/s44319-025-00563-w)
Supplement: Supplementary file 2 — Source data Fig. 1A to 1I [file 44319_2025_563_MOESM2_ESM.zip › Figure 1A-1I/Figure 1A/Figure 1A.docx]

EMBOR-2025-61534-T

Human PDAC data from The Cancer Genome Atlas from cBioPortal ([http://www.cbioportal.org](http://www.cbioportal.org/))

Individual diploid tumor samples identified as pancreatic ductal adenocarcinomas were evaluated by RNA z-score for STAT3, STAT3 response genes (signature) and KRAS dependent genes (signature).

Heatmaps display z-scores

**Figure 1A**

**Human PDAC tumor samples**

**Expression profiles**
