# Supplementary material for: STAT3 sustains tumorigenicity following mutant KRAS ablation
Source: EMBO Rep. 2025 Aug 26;26(20):4900–22. doi: 10.1038/s44319-025-00563-w (PMC12549880; doi:10.1038/s44319-025-00563-w)
Supplement: Supplementary file 2 — Source data Fig. 1A to 1I [file 44319_2025_563_MOESM2_ESM.zip › Figure 1A-1I/Figure 1G/Figure 1G.pptx]

## Slide 1
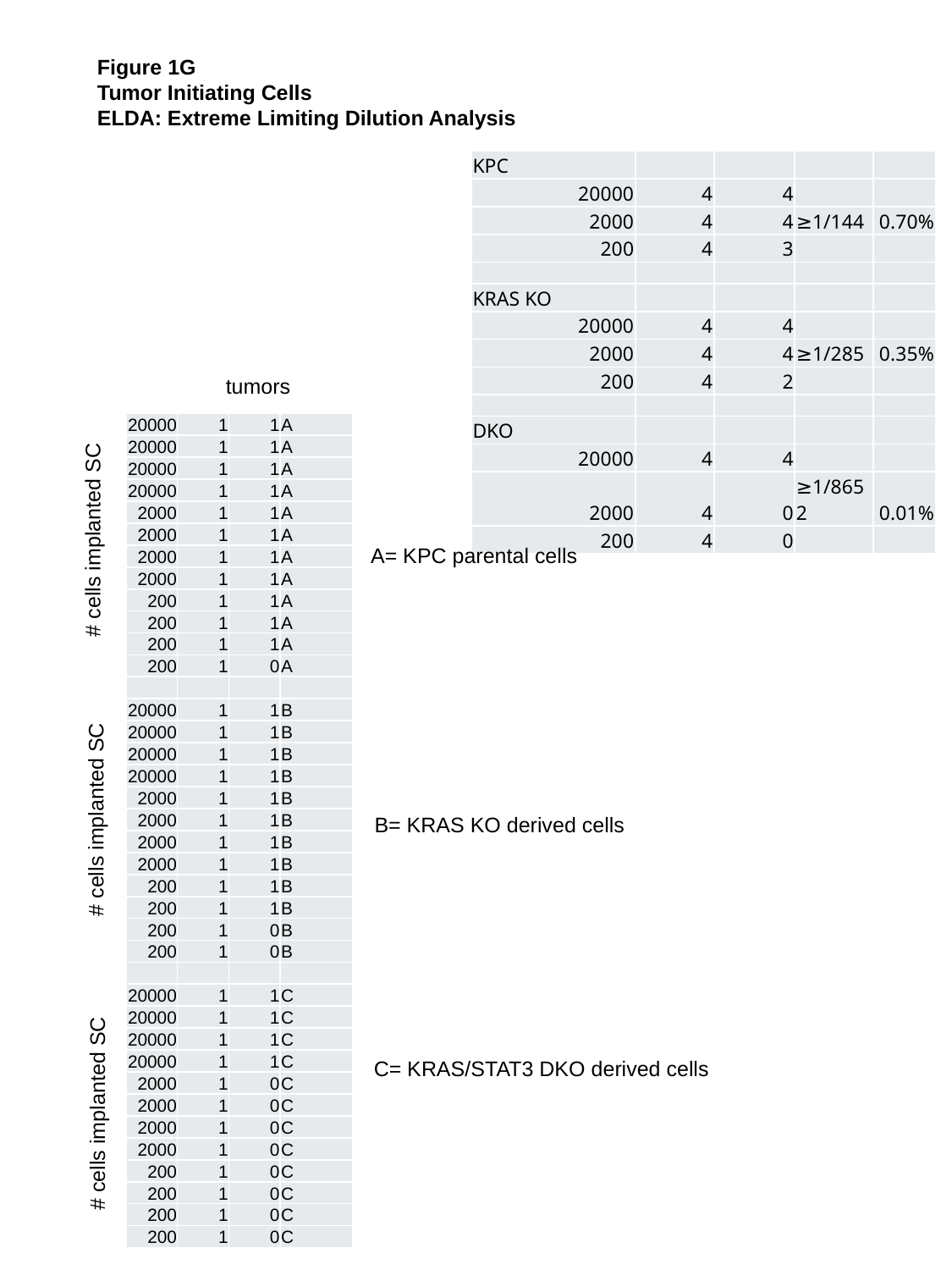

Figure 1G
Tumor Initiating Cells
ELDA: Extreme Limiting Dilution Analysis
| KPC | | | | |
| --- | --- | --- | --- | --- |
| 20000 | 4 | 4 | | |
| 2000 | 4 | 4 | ≥1/144 | 0.70% |
| 200 | 4 | 3 | | |
| | | | | |
| KRAS KO | | | | |
| 20000 | 4 | 4 | | |
| 2000 | 4 | 4 | ≥1/285 | 0.35% |
| 200 | 4 | 2 | | |
| | | | | |
| DKO | | | | |
| 20000 | 4 | 4 | | |
| 2000 | 4 | 0 | ≥1/8652 | 0.01% |
| 200 | 4 | 0 | | |
tumors
| 20000 | 1 | 1 | A |
| --- | --- | --- | --- |
| 20000 | 1 | 1 | A |
| 20000 | 1 | 1 | A |
| 20000 | 1 | 1 | A |
| 2000 | 1 | 1 | A |
| 2000 | 1 | 1 | A |
| 2000 | 1 | 1 | A |
| 2000 | 1 | 1 | A |
| 200 | 1 | 1 | A |
| 200 | 1 | 1 | A |
| 200 | 1 | 1 | A |
| 200 | 1 | 0 | A |
| | | | |
| 20000 | 1 | 1 | B |
| 20000 | 1 | 1 | B |
| 20000 | 1 | 1 | B |
| 20000 | 1 | 1 | B |
| 2000 | 1 | 1 | B |
| 2000 | 1 | 1 | B |
| 2000 | 1 | 1 | B |
| 2000 | 1 | 1 | B |
| 200 | 1 | 1 | B |
| 200 | 1 | 1 | B |
| 200 | 1 | 0 | B |
| 200 | 1 | 0 | B |
| | | | |
| 20000 | 1 | 1 | C |
| 20000 | 1 | 1 | C |
| 20000 | 1 | 1 | C |
| 20000 | 1 | 1 | C |
| 2000 | 1 | 0 | C |
| 2000 | 1 | 0 | C |
| 2000 | 1 | 0 | C |
| 2000 | 1 | 0 | C |
| 200 | 1 | 0 | C |
| 200 | 1 | 0 | C |
| 200 | 1 | 0 | C |
| 200 | 1 | 0 | C |
# cells implanted SC
A= KPC parental cells
# cells implanted SC
B= KRAS KO derived cells
C= KRAS/STAT3 DKO derived cells
# cells implanted SC
