# Supplementary material for: STAT3 sustains tumorigenicity following mutant KRAS ablation
Source: EMBO Rep. 2025 Aug 26;26(20):4900–22. doi: 10.1038/s44319-025-00563-w (PMC12549880; doi:10.1038/s44319-025-00563-w)
Supplement: Supplementary file 2 — Source data Fig. 1A to 1I [file 44319_2025_563_MOESM2_ESM.zip › Figure 1A-1I/Figure 1E/Figure 1E.pptx]

## Slide 1
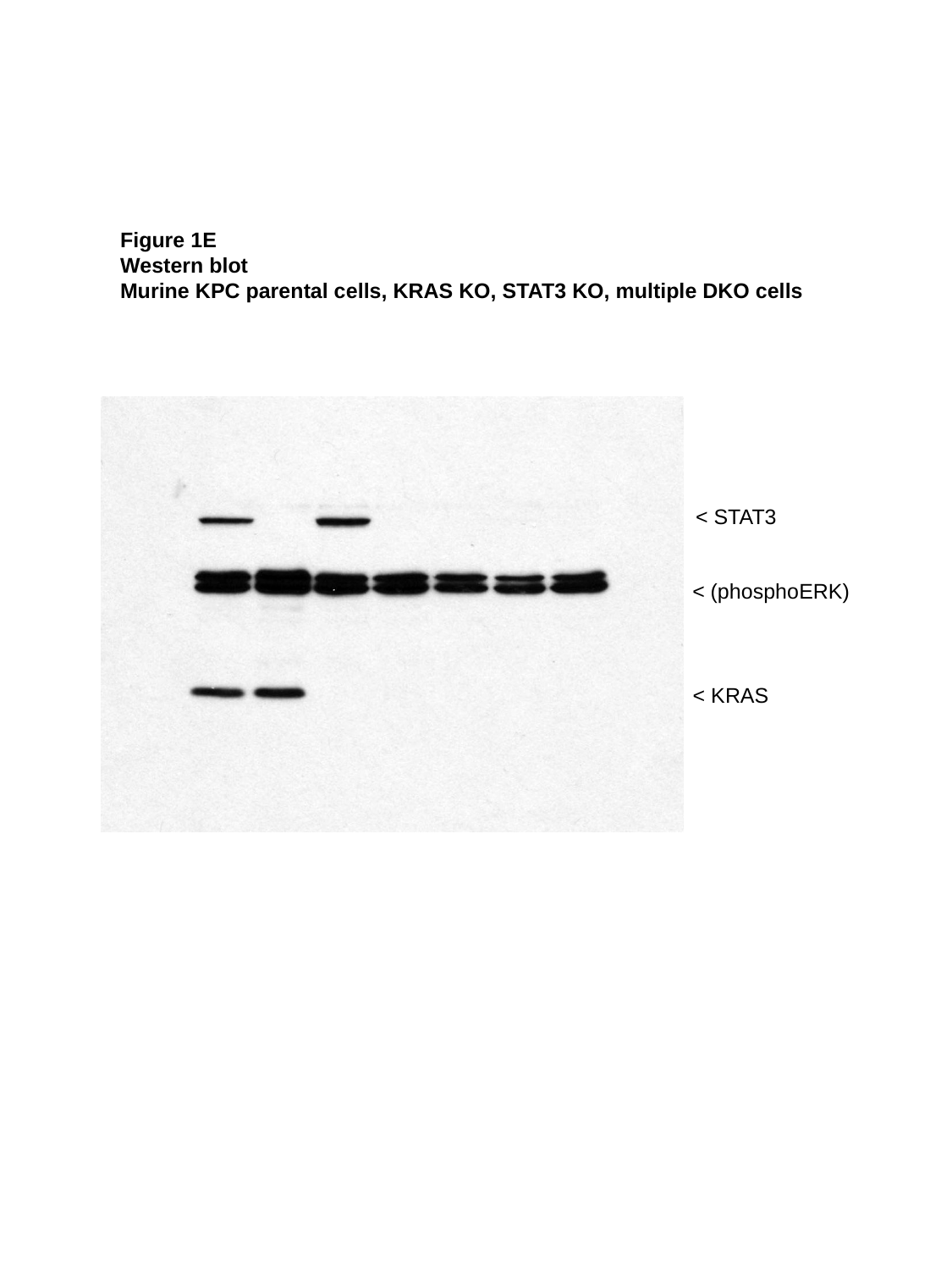

Figure 1E
Western blot
Murine KPC parental cells, KRAS KO, STAT3 KO, multiple DKO cells
< STAT3
< (phosphoERK)
< KRAS
