# Supplementary material for: STAT3 sustains tumorigenicity following mutant KRAS ablation
Source: EMBO Rep. 2025 Aug 26;26(20):4900–22. doi: 10.1038/s44319-025-00563-w (PMC12549880; doi:10.1038/s44319-025-00563-w)
Supplement: Supplementary file 2 — Source data Fig. 1A to 1I [file 44319_2025_563_MOESM2_ESM.zip › Figure 1A-1I/Figure 1C/Figure 1C.pptx]

## Slide 1
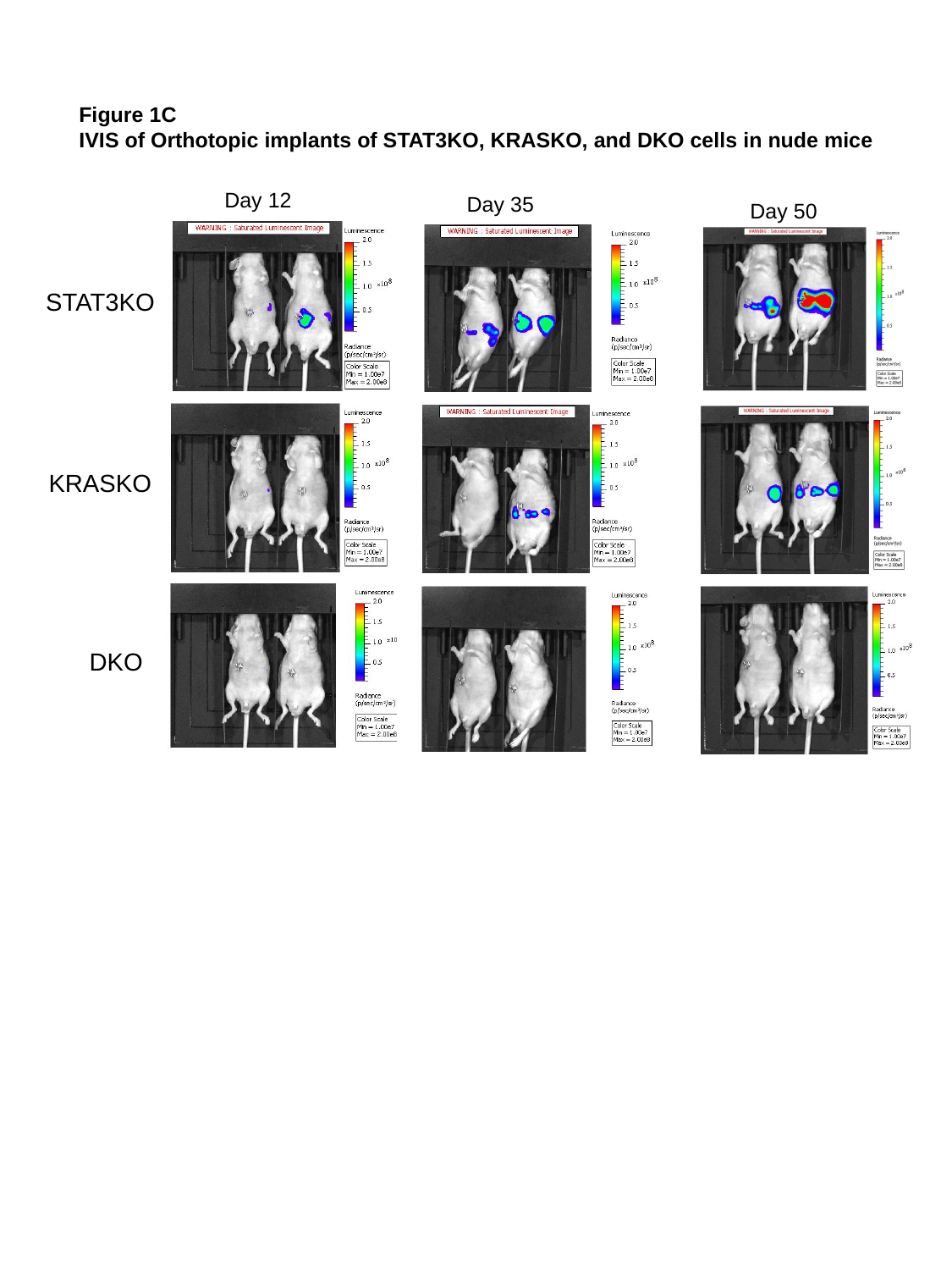

Figure 1C
IVIS of Orthotopic implants of STAT3KO, KRASKO, and DKO cells in nude mice
Day 12
Day 35
Day 50
STAT3KO
KRASKO
DKO
