# Supplementary figures and images for: STAT3 sustains tumorigenicity following mutant KRAS ablation
Source: EMBO Rep. 2025 Aug 26;26(20):4900–22. doi: 10.1038/s44319-025-00563-w (PMC12549880; doi:10.1038/s44319-025-00563-w)

## Slide 1
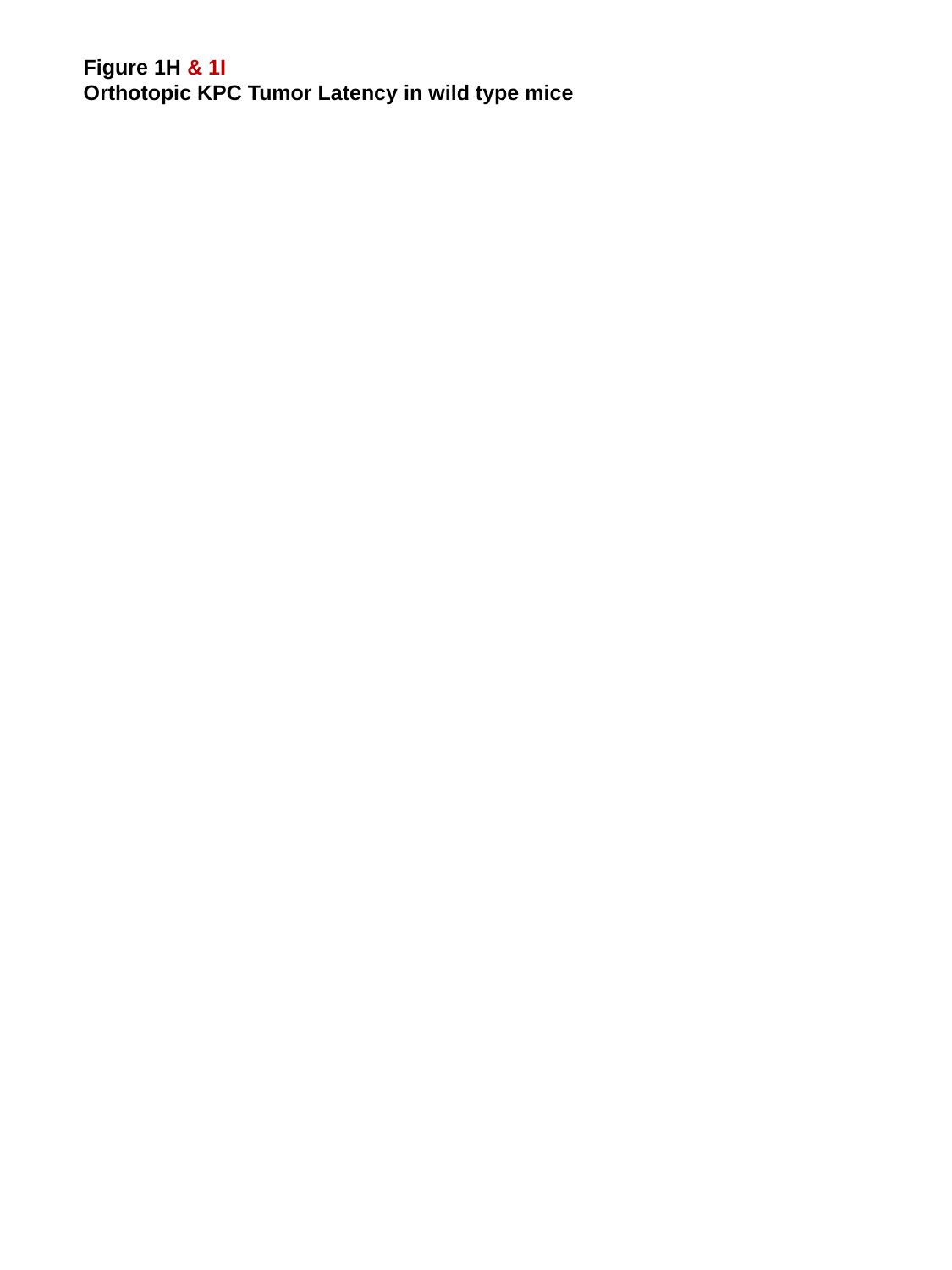

Figure 1H & 1I
Orthotopic KPC Tumor Latency in wild type mice

Supplement: Supplementary file 2 — Source data Fig. 1A to 1I [file 44319_2025_563_MOESM2_ESM.zip › Figure 1A-1I/Figure 1H/Figure 1H.pptx]

## Slide 1
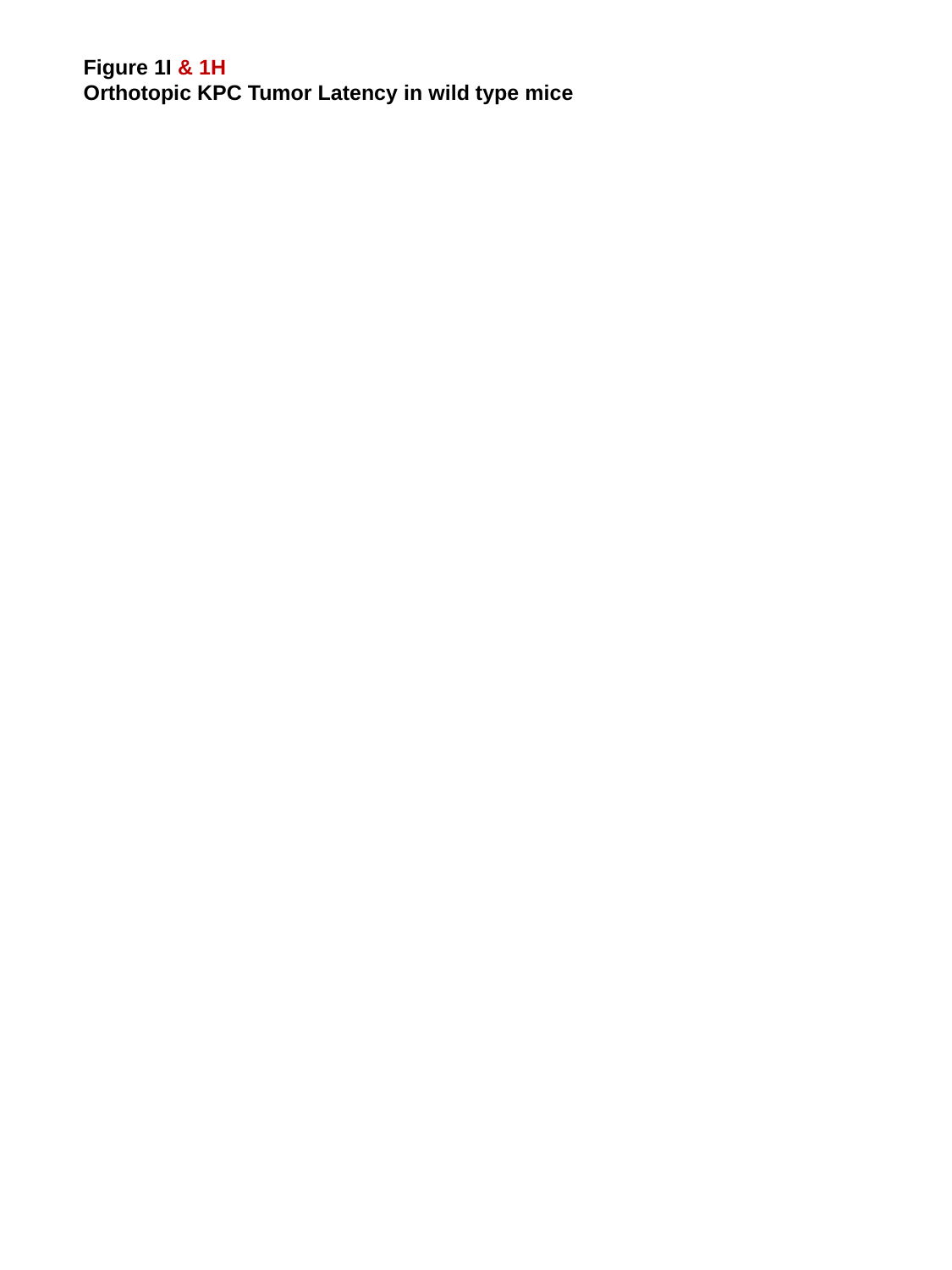

Figure 1I & 1H
Orthotopic KPC Tumor Latency in wild type mice

Supplement: Supplementary file 2 — Source data Fig. 1A to 1I [file 44319_2025_563_MOESM2_ESM.zip › Figure 1A-1I/Figure 1I/Figure 1I.pptx]

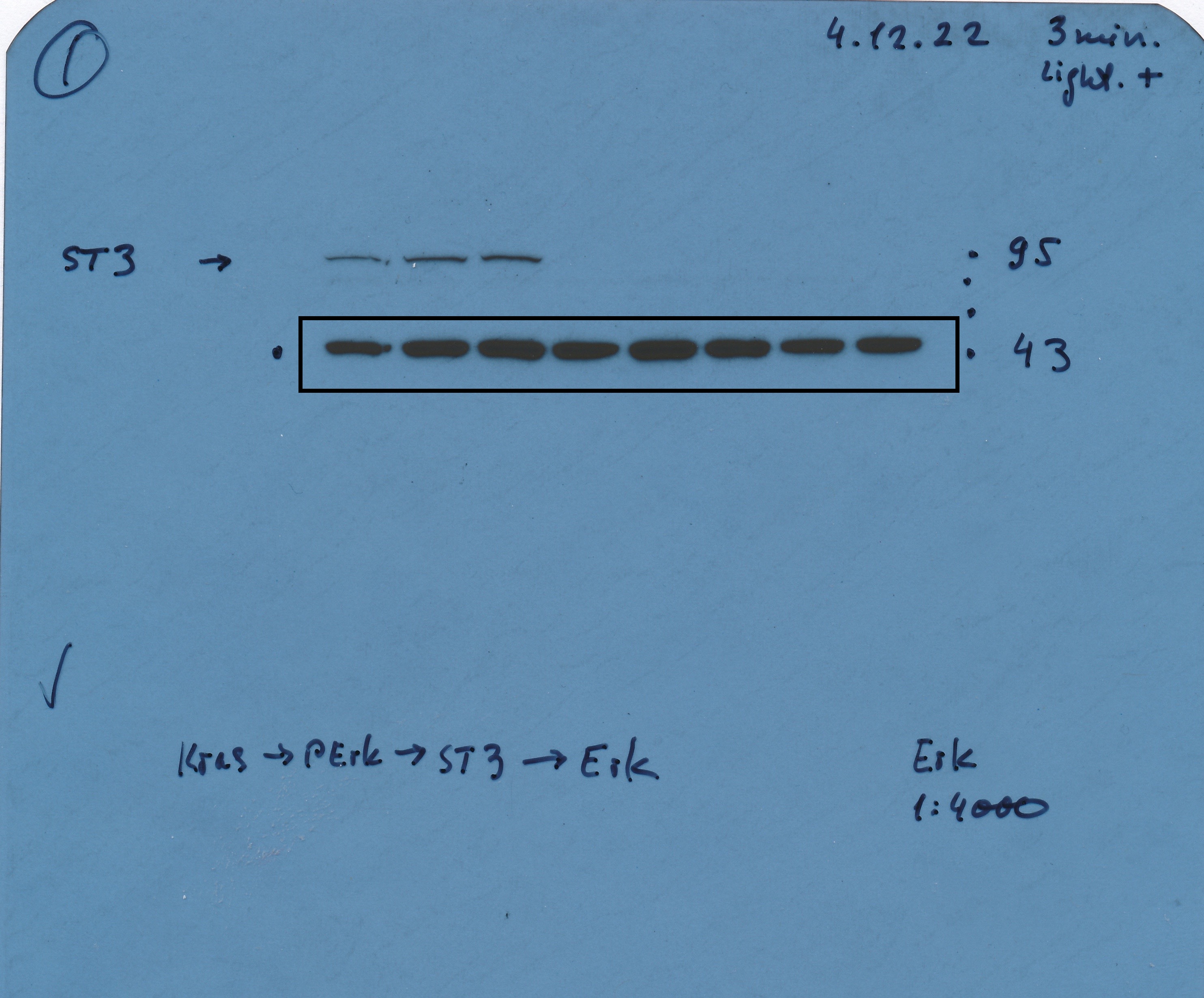

Supplement: Supplementary file 2 — Source data Fig. 1A to 1I [file 44319_2025_563_MOESM2_ESM.zip › Figure 1A-1I/Figure 1B/2022-04-12_Panc_gel1_Erk (1).jpg]

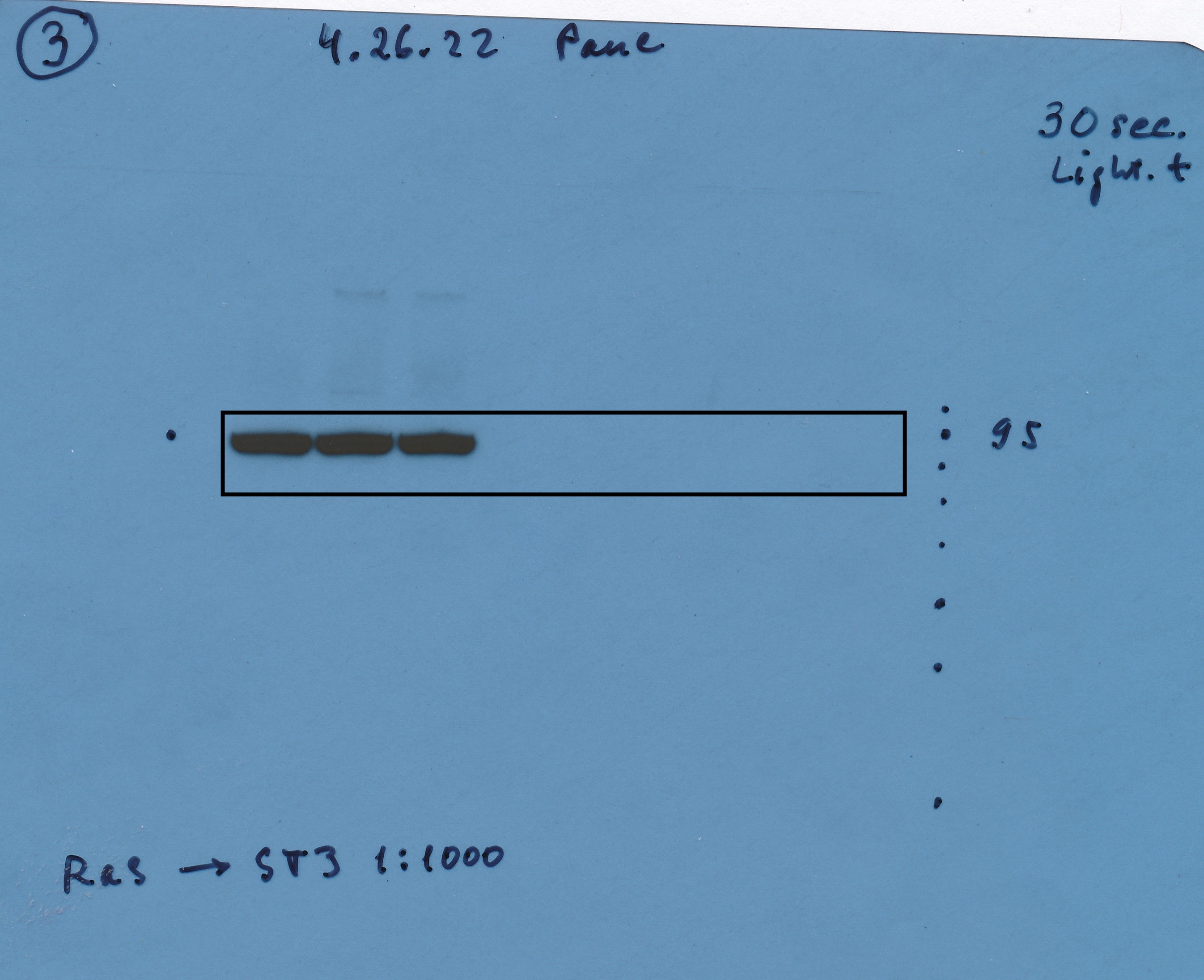

Supplement: Supplementary file 2 — Source data Fig. 1A to 1I [file 44319_2025_563_MOESM2_ESM.zip › Figure 1A-1I/Figure 1B/2022-04-26_Panc_gel3_ST3 (1).jpg]

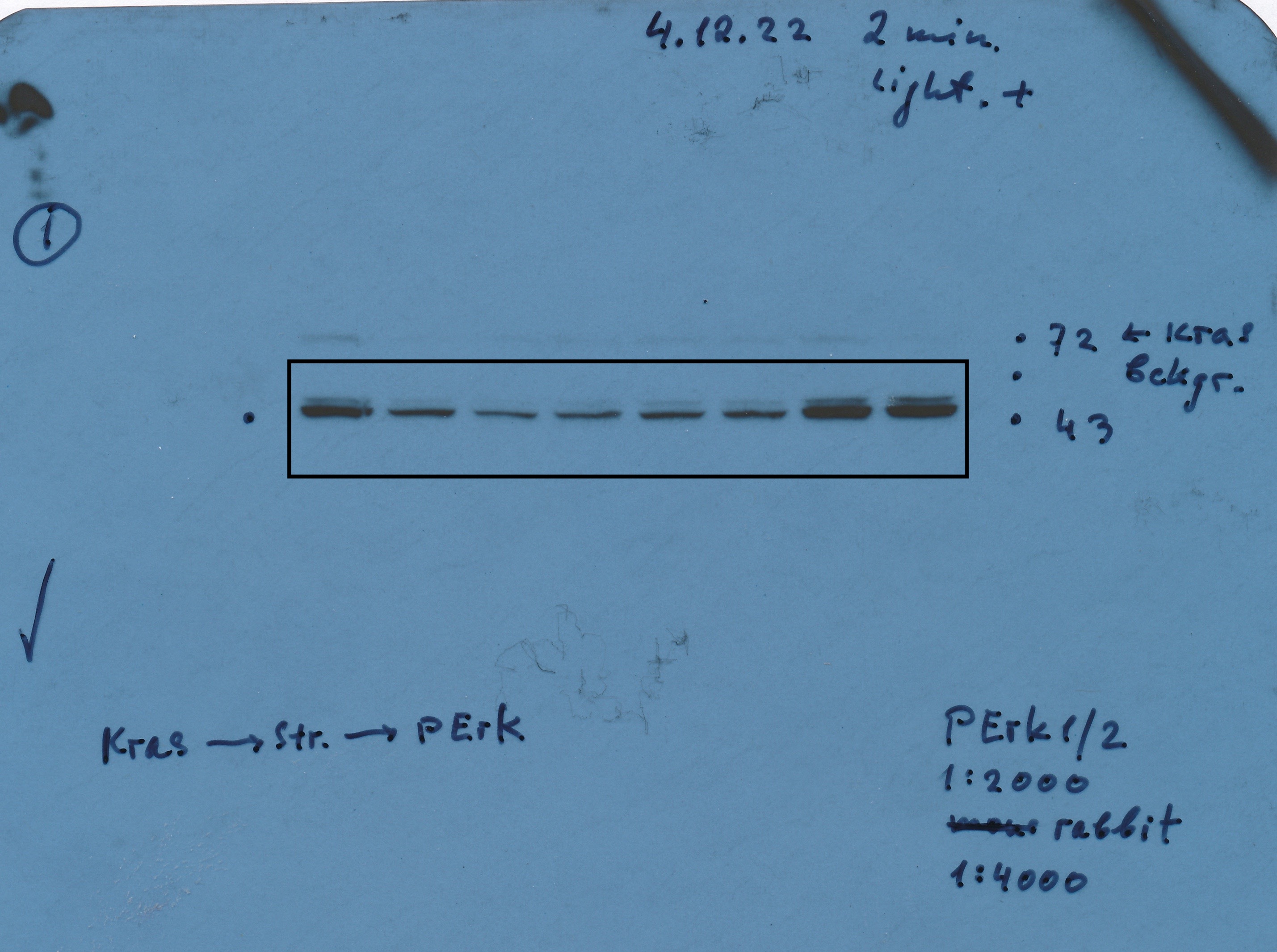

Supplement: Supplementary file 2 — Source data Fig. 1A to 1I [file 44319_2025_563_MOESM2_ESM.zip › Figure 1A-1I/Figure 1B/2022-04-12_Panc_gel1_PErk (1).jpg]

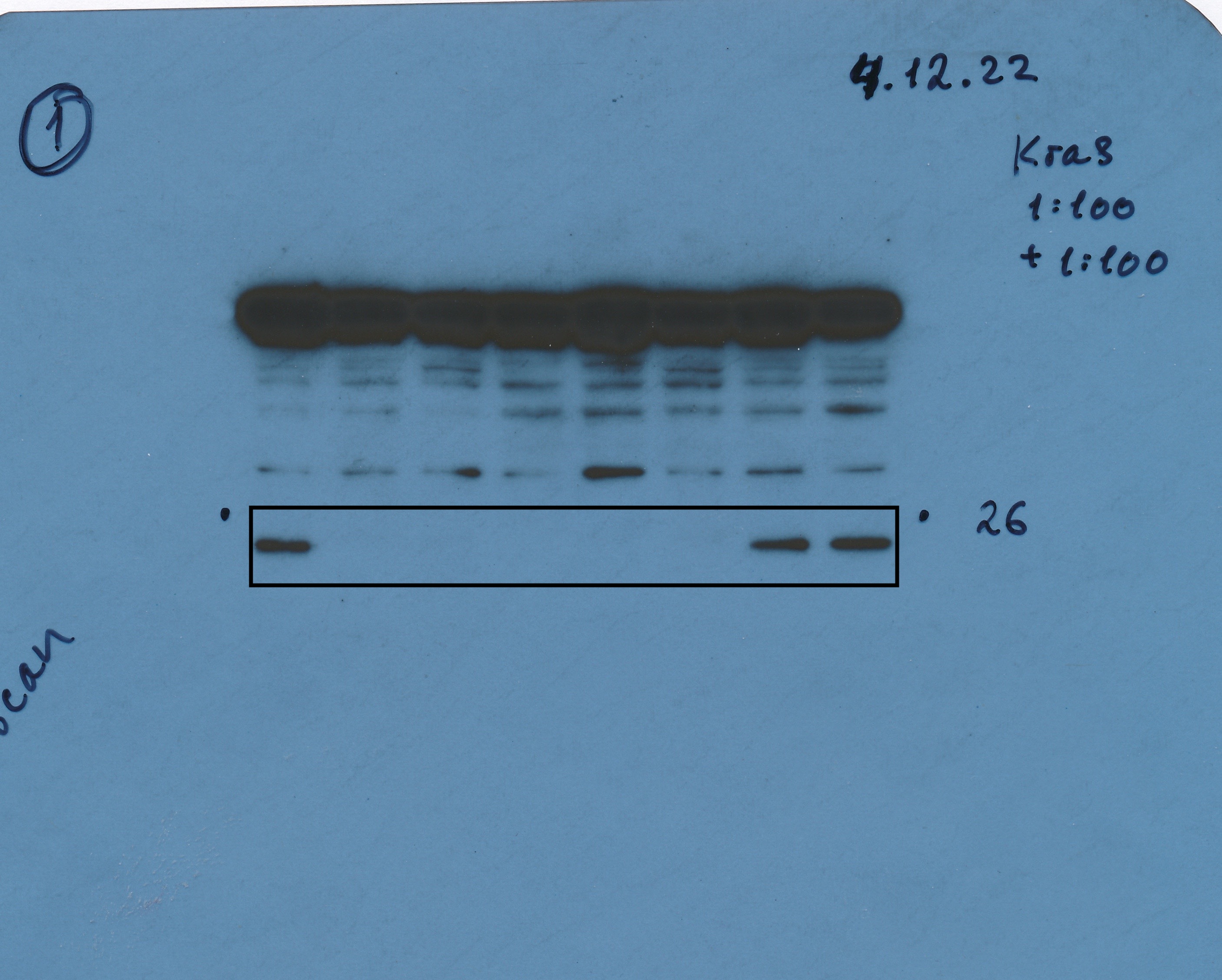

Supplement: Supplementary file 2 — Source data Fig. 1A to 1I [file 44319_2025_563_MOESM2_ESM.zip › Figure 1A-1I/Figure 1B/2022-04-12_Panc_gel1_Kras (1).jpg]

## Slide 1
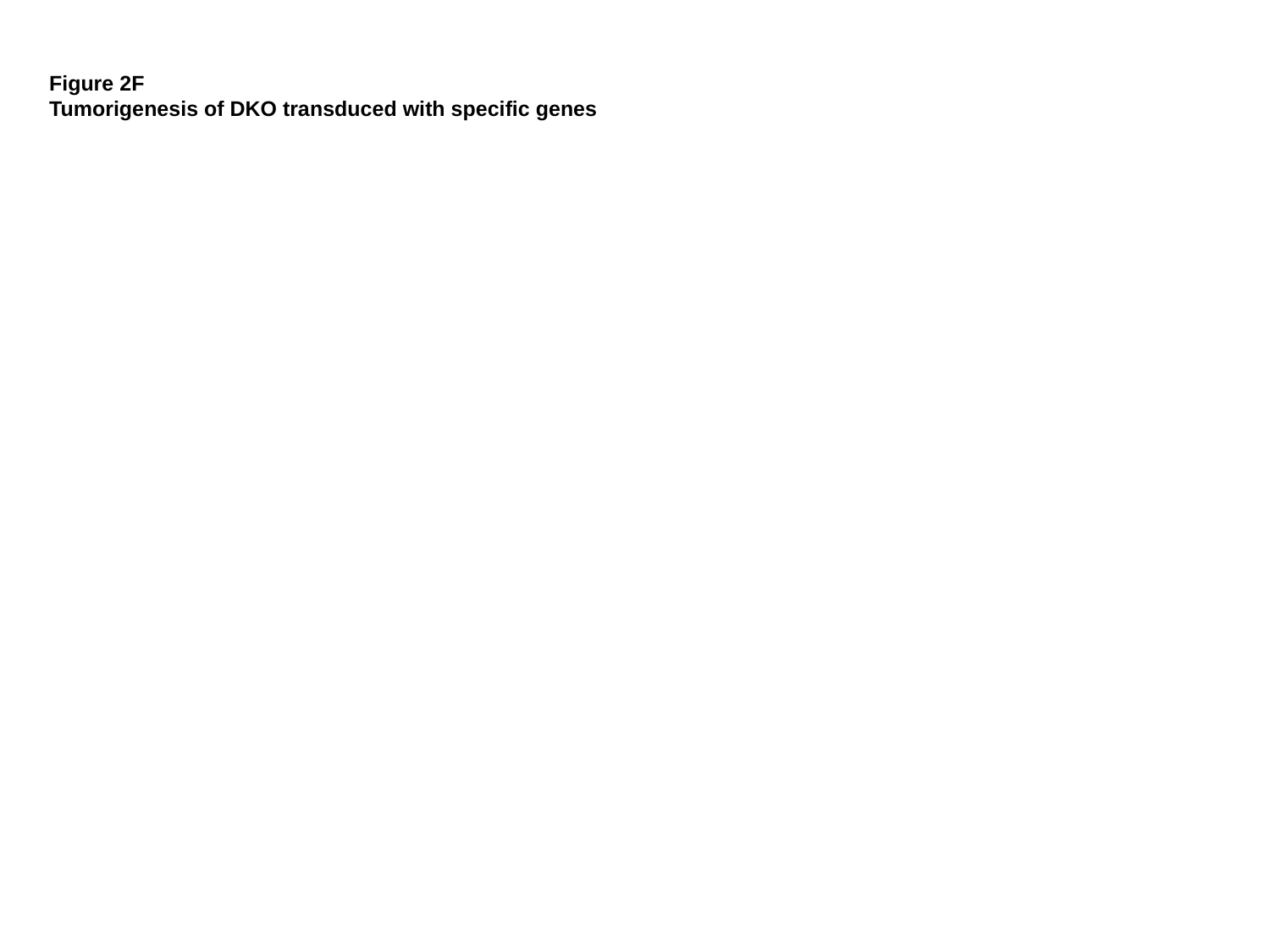

Figure 2F
Tumorigenesis of DKO transduced with specific genes

Supplement: Supplementary file 4 — Source data Fig. 2 [file 44319_2025_563_MOESM4_ESM.zip › Figure 2/Figure 2F/Figure 2F.pptx]

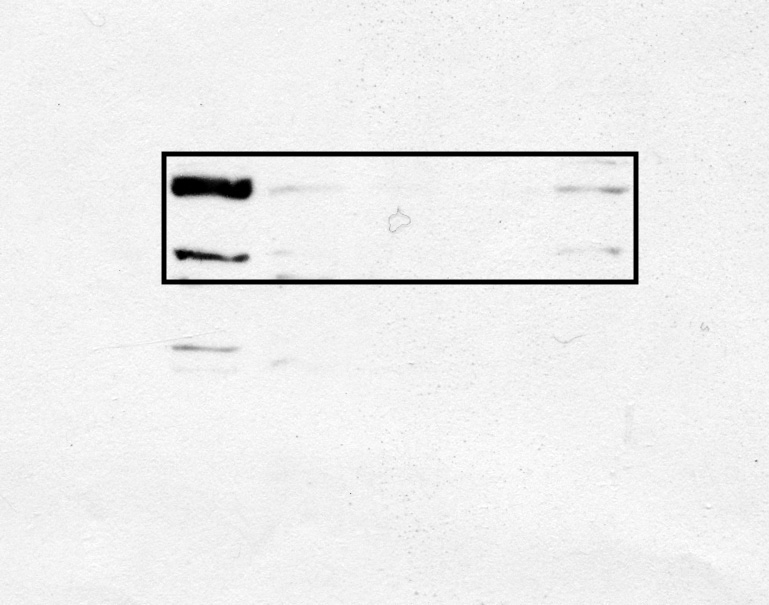

Supplement: Supplementary file 4 — Source data Fig. 2 [file 44319_2025_563_MOESM4_ESM.zip › Figure 2/Figure 2E/B6 + DKOs HNF4A redo 81220.jpg]

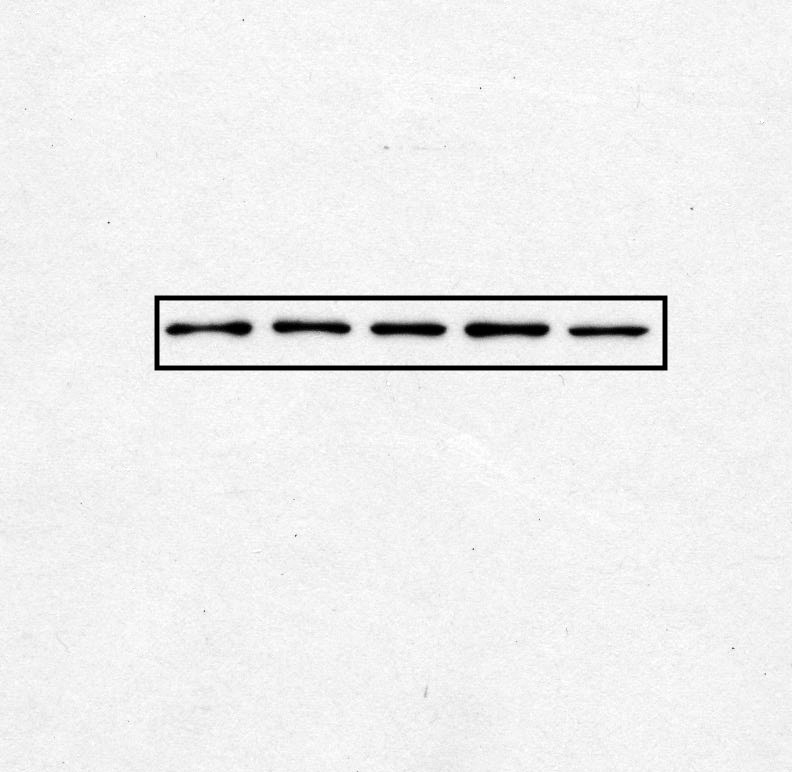

Supplement: Supplementary file 4 — Source data Fig. 2 [file 44319_2025_563_MOESM4_ESM.zip › Figure 2/Figure 2E/B6 + DKOs total ERK revised 31320.jpg]

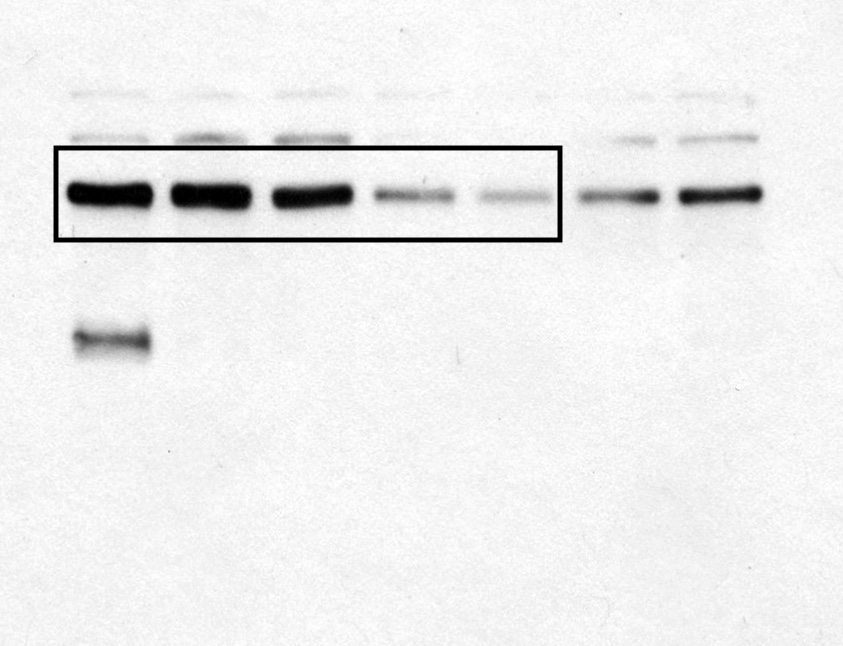

Supplement: Supplementary file 4 — Source data Fig. 2 [file 44319_2025_563_MOESM4_ESM.zip › Figure 2/Figure 2E/B6 + DKOs + 640 KLF5.jpg]

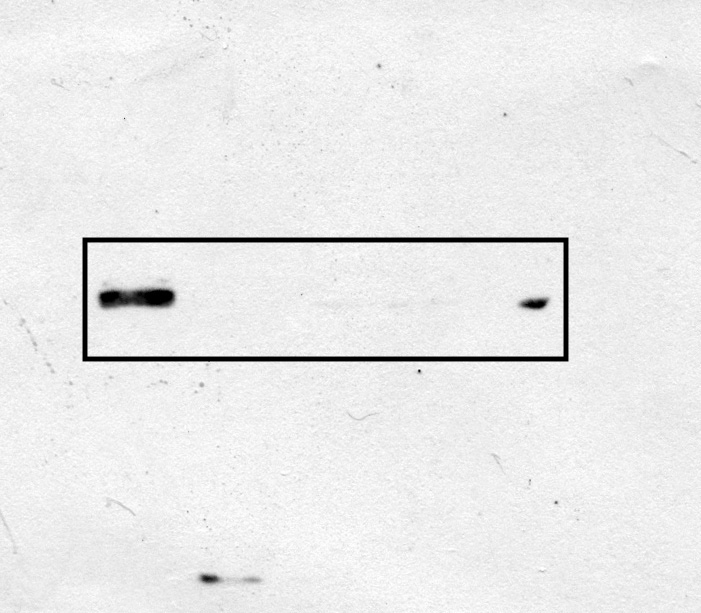

Supplement: Supplementary file 4 — Source data Fig. 2 [file 44319_2025_563_MOESM4_ESM.zip › Figure 2/Figure 2E/B6 + DKOs KLF4 81220.jpg]

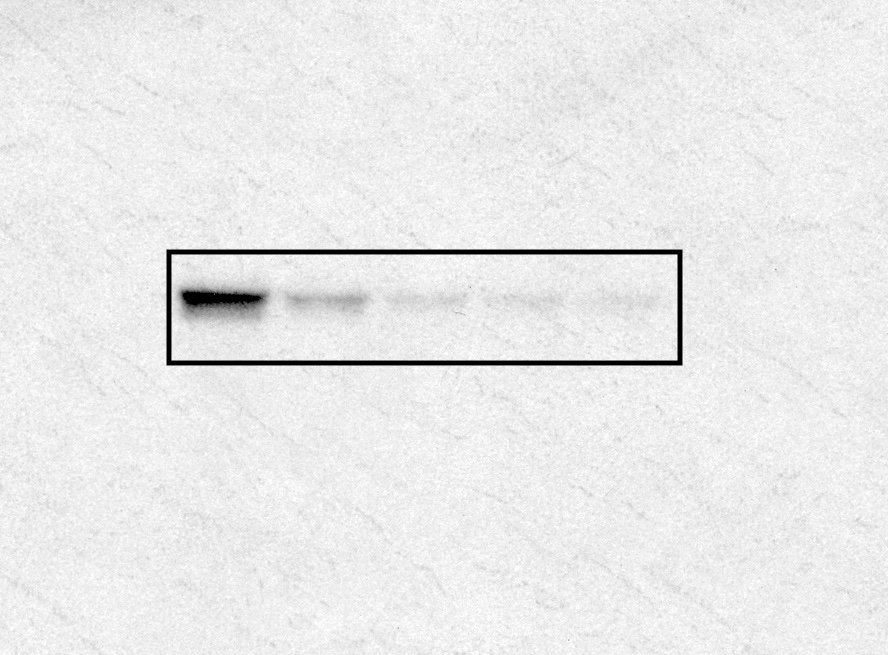

Supplement: Supplementary file 4 — Source data Fig. 2 [file 44319_2025_563_MOESM4_ESM.zip › Figure 2/Figure 2E/B6 + DKOs FOXA2 71720.jpg]

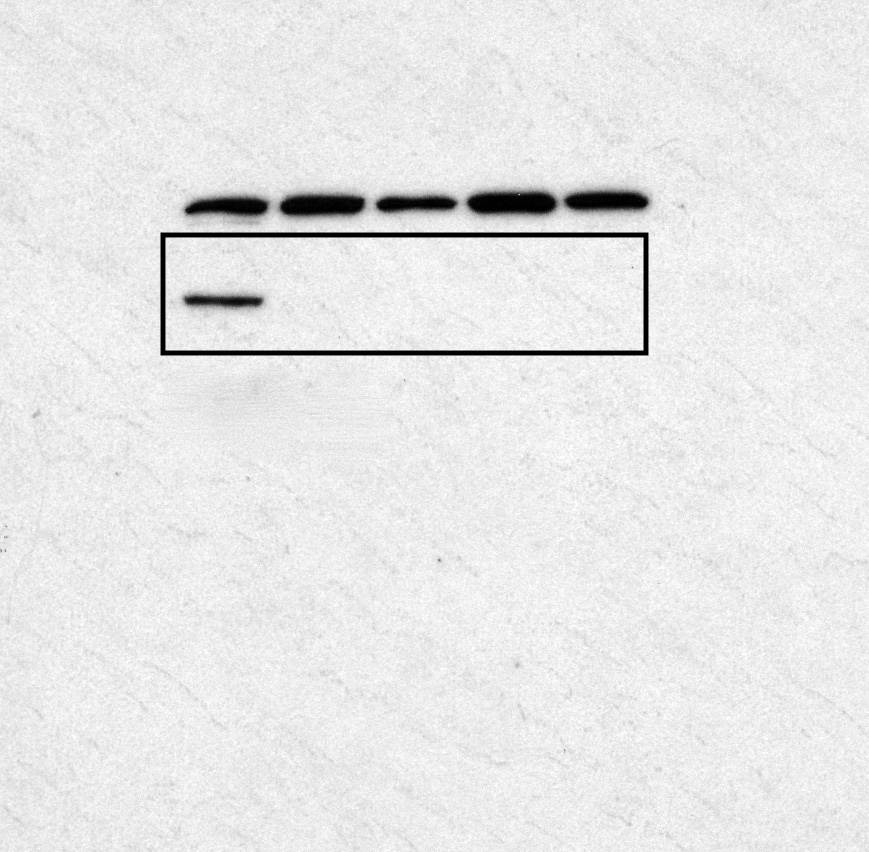

Supplement: Supplementary file 4 — Source data Fig. 2 [file 44319_2025_563_MOESM4_ESM.zip › Figure 2/Figure 2E/B6 + DKOs FOXA1 72320.jpg]

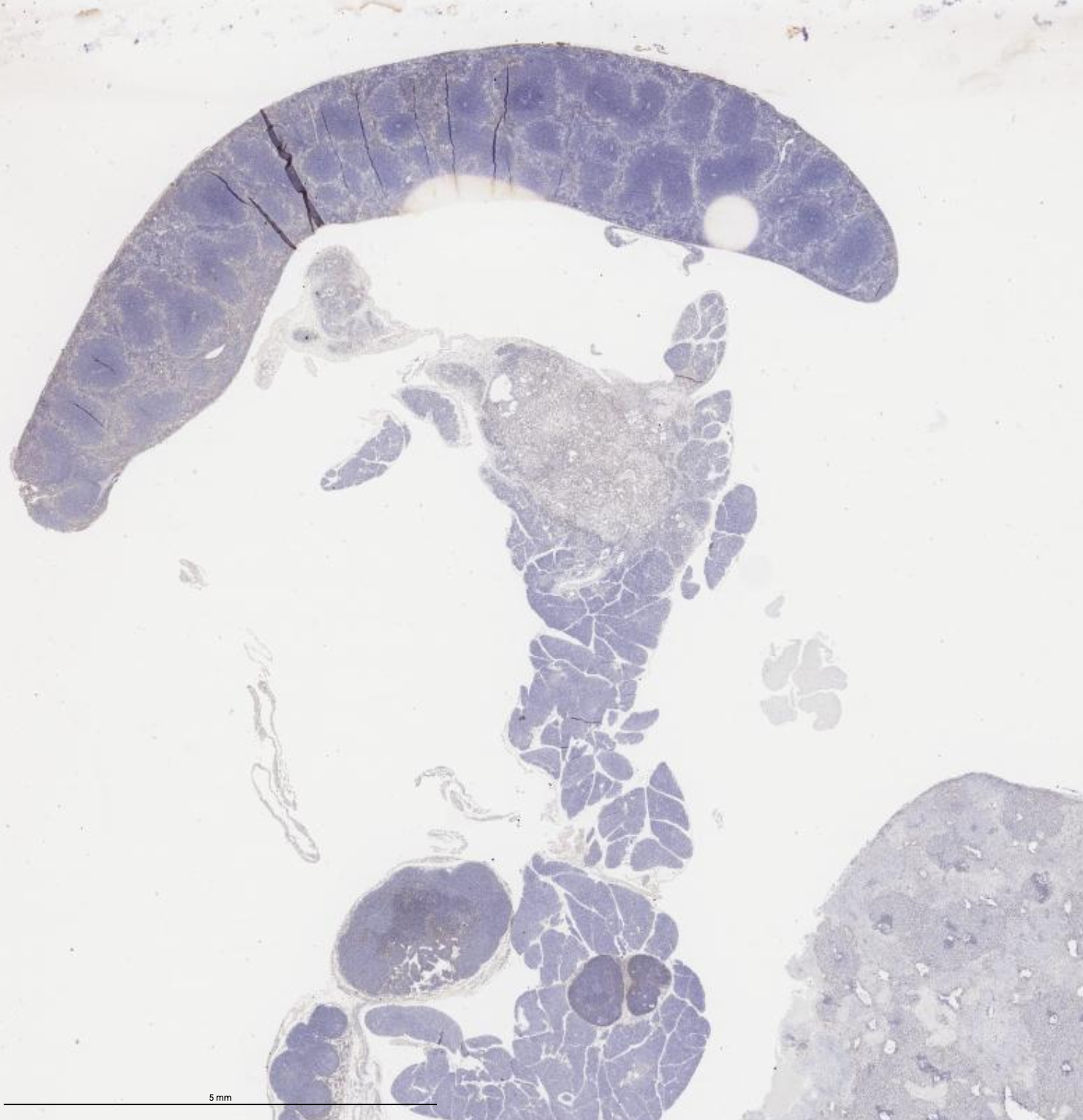

Supplement: Supplementary file 5 — Source data Fig. 3 [file 44319_2025_563_MOESM5_ESM.zip › Figure 3/Figure 3A/KPC 5 Mouse Treated anti-PYSTAT3_.png]

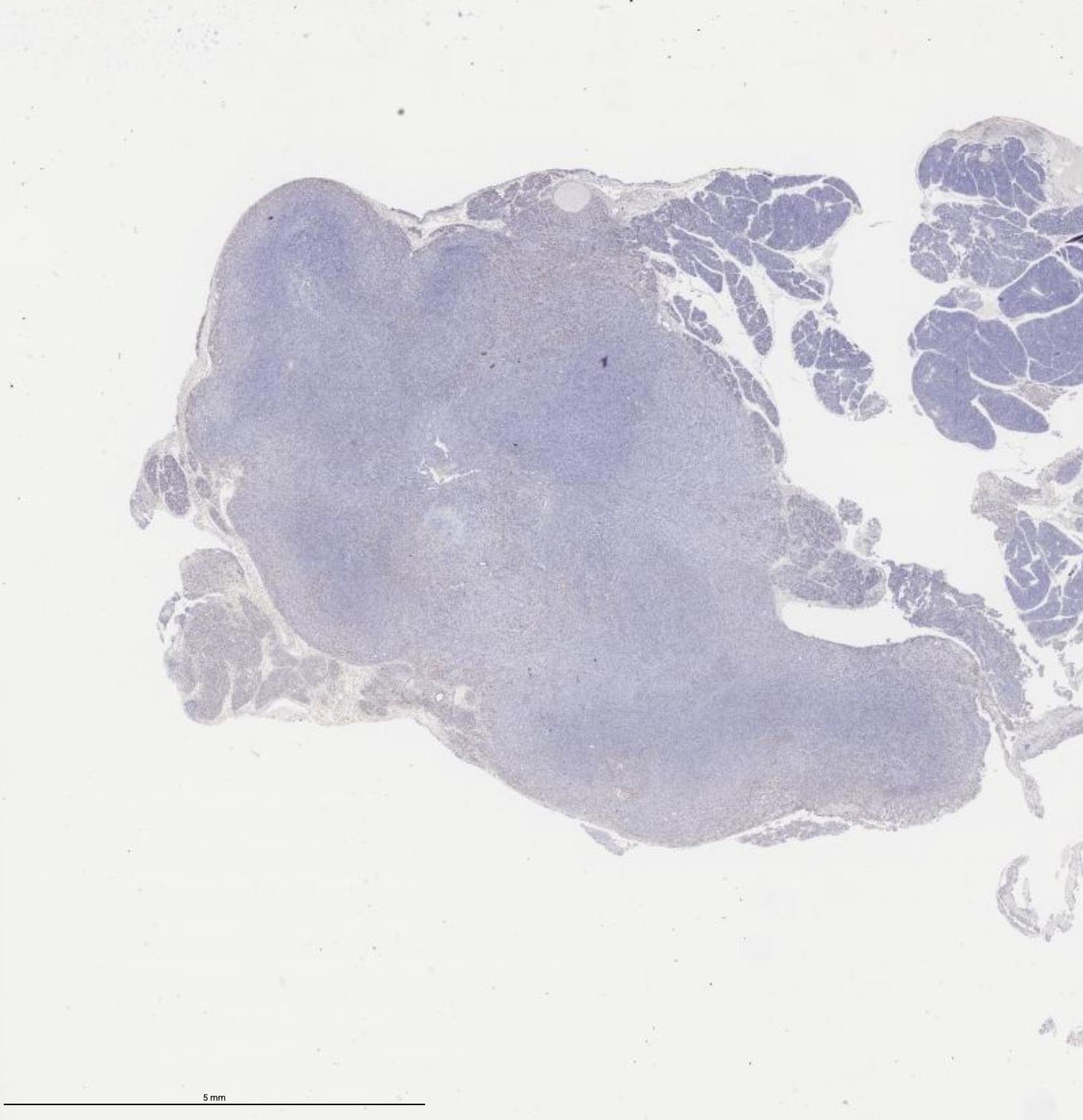

Supplement: Supplementary file 5 — Source data Fig. 3 [file 44319_2025_563_MOESM5_ESM.zip › Figure 3/Figure 3A/KPC 5 Mouse anti-PYSTAT3.png]

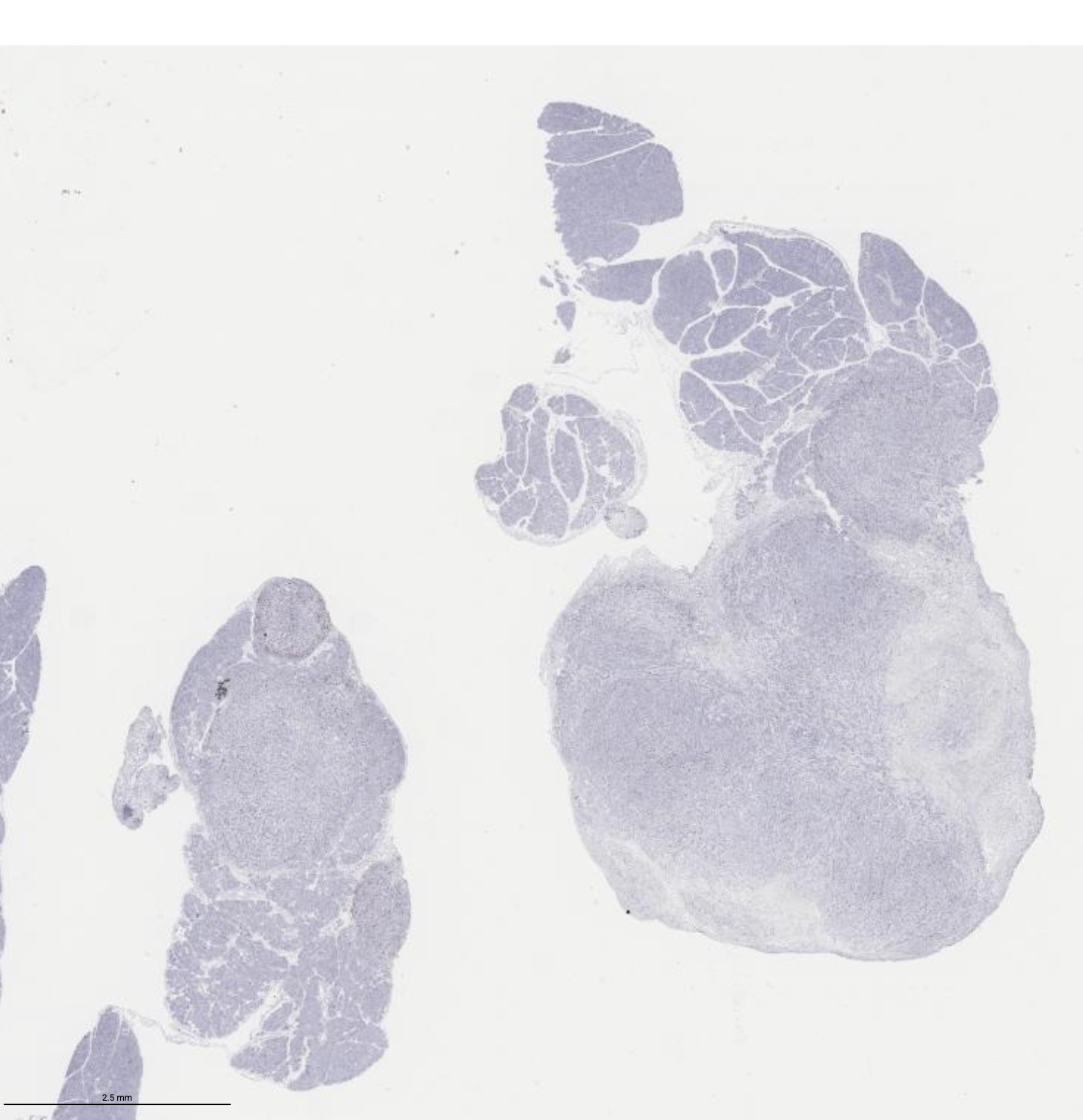

Supplement: Supplementary file 5 — Source data Fig. 3 [file 44319_2025_563_MOESM5_ESM.zip › Figure 3/Figure 3A/KPC B6 anti-CD8a_.png]

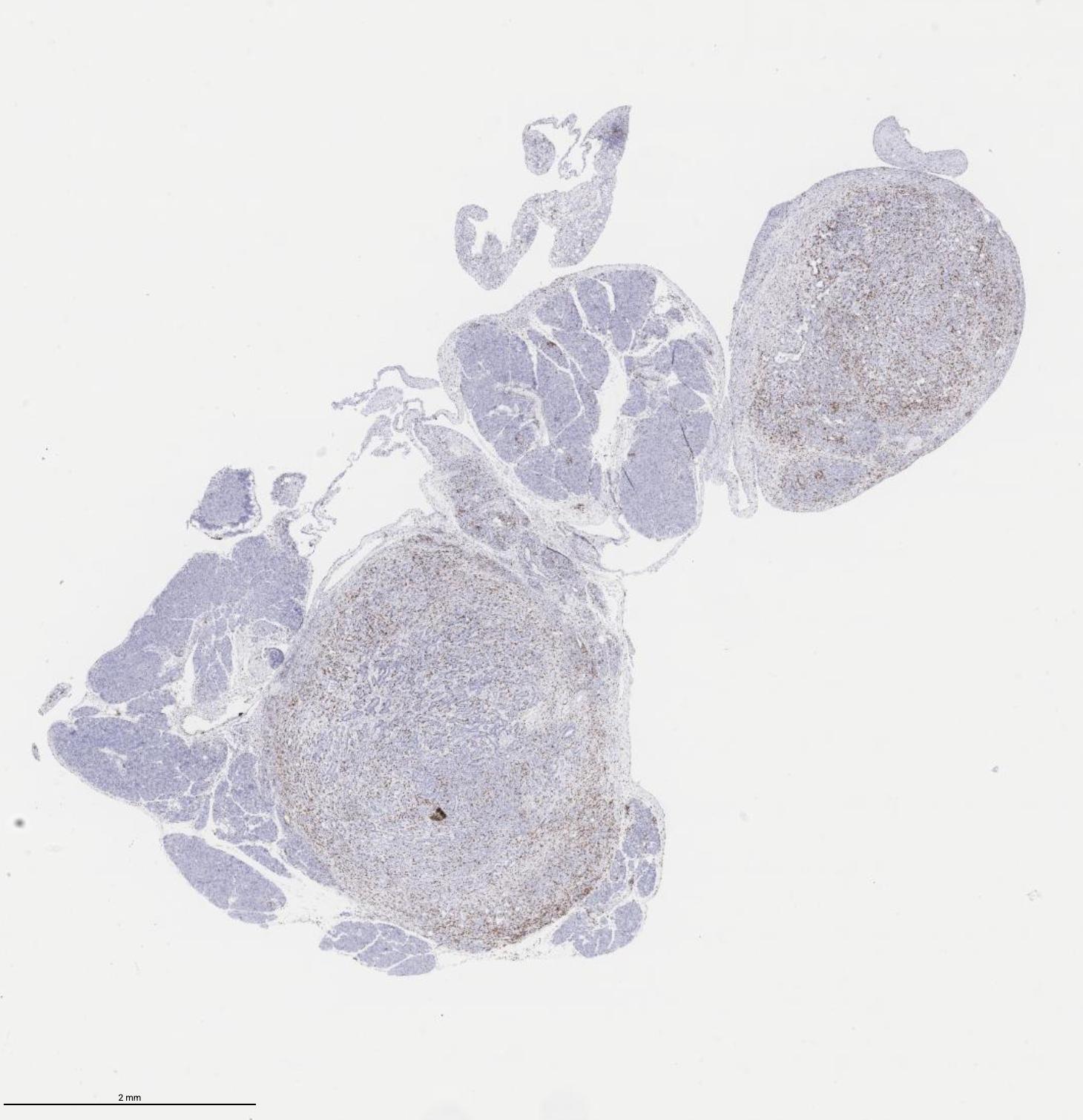

Supplement: Supplementary file 5 — Source data Fig. 3 [file 44319_2025_563_MOESM5_ESM.zip › Figure 3/Figure 3A/KPC B6 Mouse Treated anti-CD8a_.png]

## Slide 1
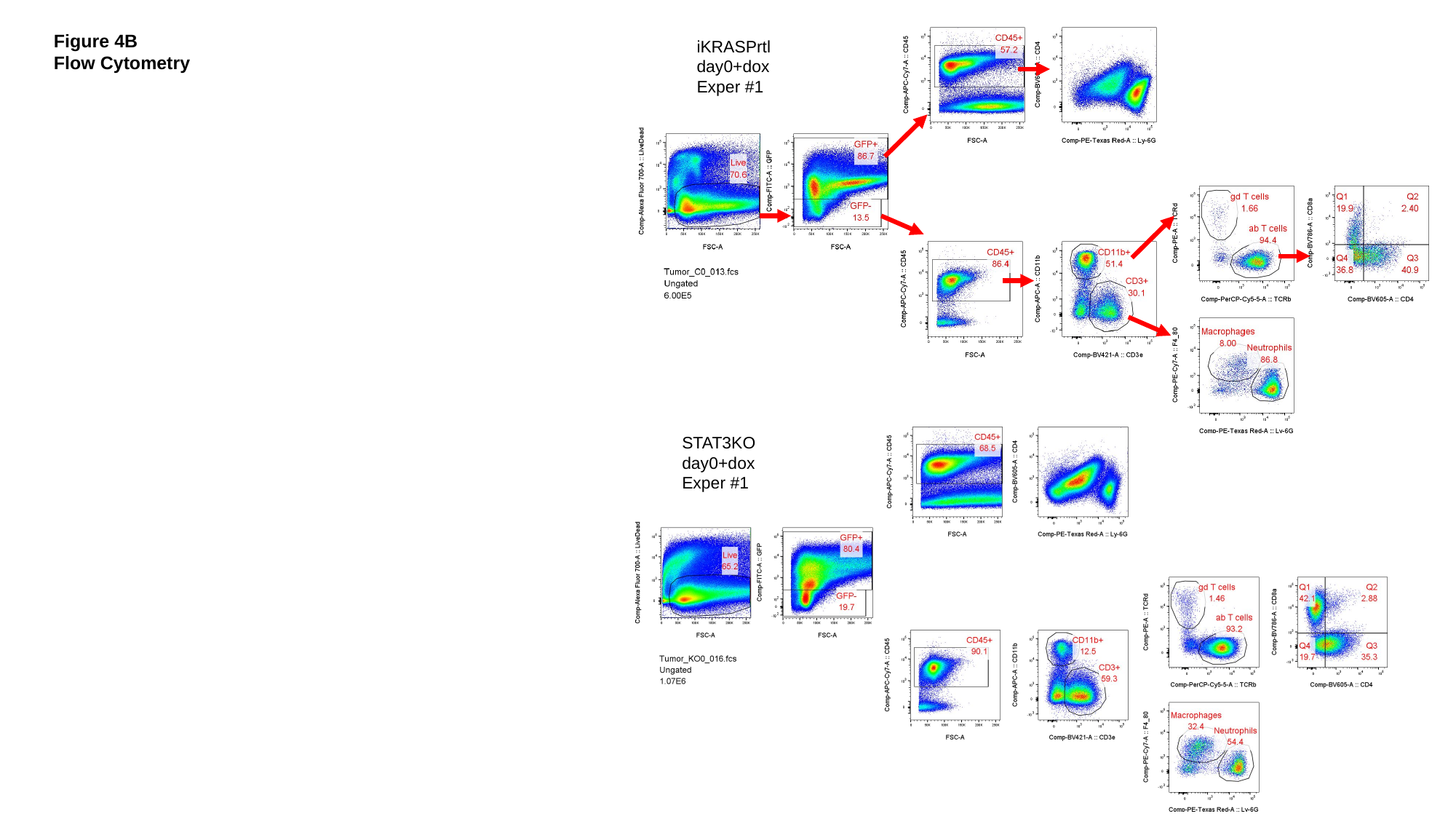

iKRASPrtl day0+dox
Exper #1
Figure 4B
Flow Cytometry
STAT3KO day0+dox
Exper #1

Supplement: Supplementary file 6 — Source data Fig. 4 [file 44319_2025_563_MOESM6_ESM.zip › Figure 4/Figure 4B/Figure 4B.pptx]

## Slide 1
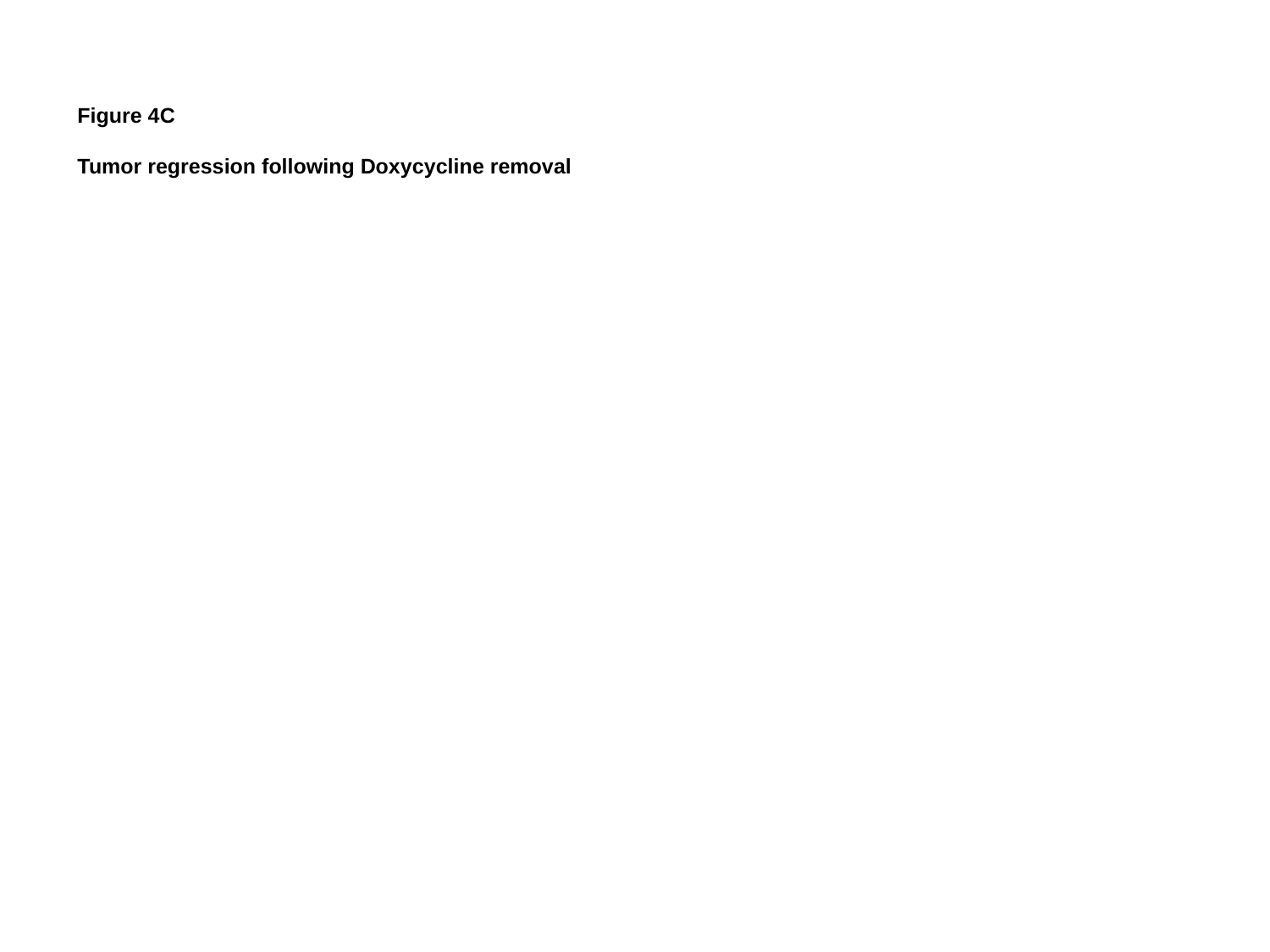

Figure 4C
Tumor regression following Doxycycline removal

Supplement: Supplementary file 6 — Source data Fig. 4 [file 44319_2025_563_MOESM6_ESM.zip › Figure 4/Figure 4C/Figure 4C.pptx]

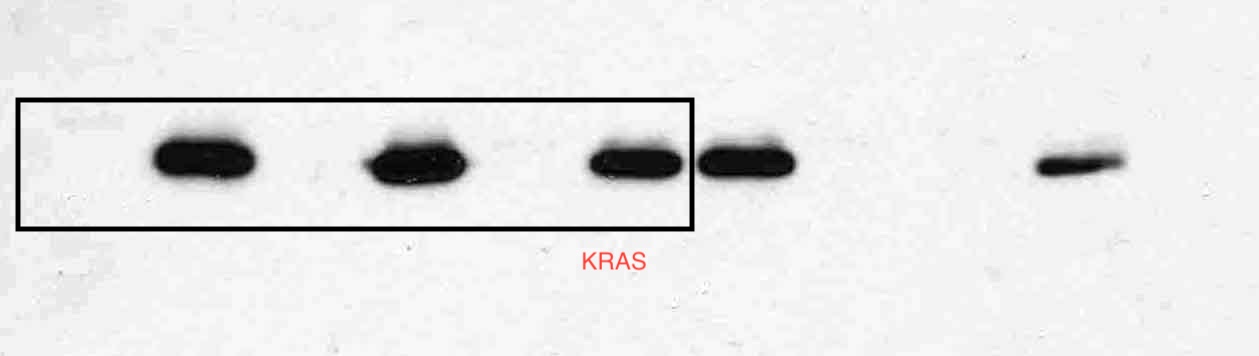

Supplement: Supplementary file 6 — Source data Fig. 4 [file 44319_2025_563_MOESM6_ESM.zip › Figure 4/Figure 4A/iKRAS_STAT3KO clones -_+ Doxycycline_anti-KRAS.jpg]

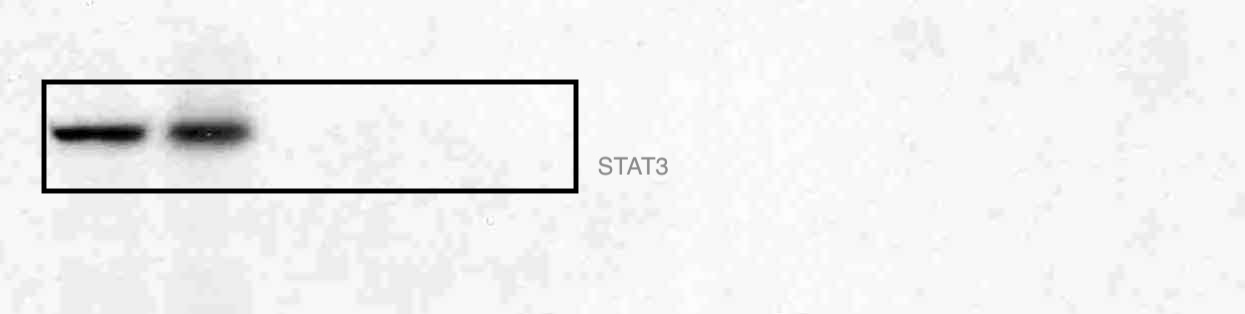

Supplement: Supplementary file 6 — Source data Fig. 4 [file 44319_2025_563_MOESM6_ESM.zip › Figure 4/Figure 4A/iKRAS_STAT3KO clones + Doxycycline_anti-STAT3 .jpg]

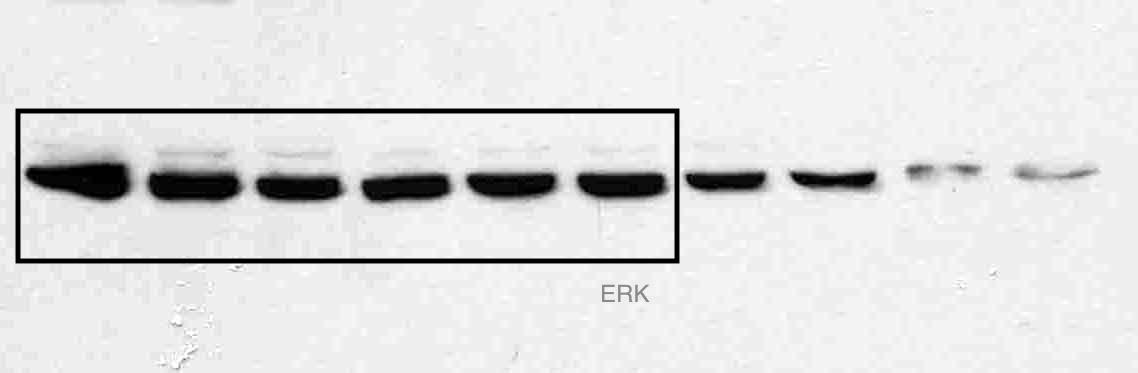

Supplement: Supplementary file 6 — Source data Fig. 4 [file 44319_2025_563_MOESM6_ESM.zip › Figure 4/Figure 4A/iKRAS_STAT3KO clones + Doxycycline_ERK.jpg]
