# Supplementary material for: STAT3 sustains tumorigenicity following mutant KRAS ablation
Source: EMBO Rep. 2025 Aug 26;26(20):4900–22. doi: 10.1038/s44319-025-00563-w (PMC12549880; doi:10.1038/s44319-025-00563-w)
Supplement: Supplementary file 4 — Source data Fig. 2 [file 44319_2025_563_MOESM4_ESM.zip › Figure 2/Figure 2G/Figure 2G.pptx]

## Slide 1
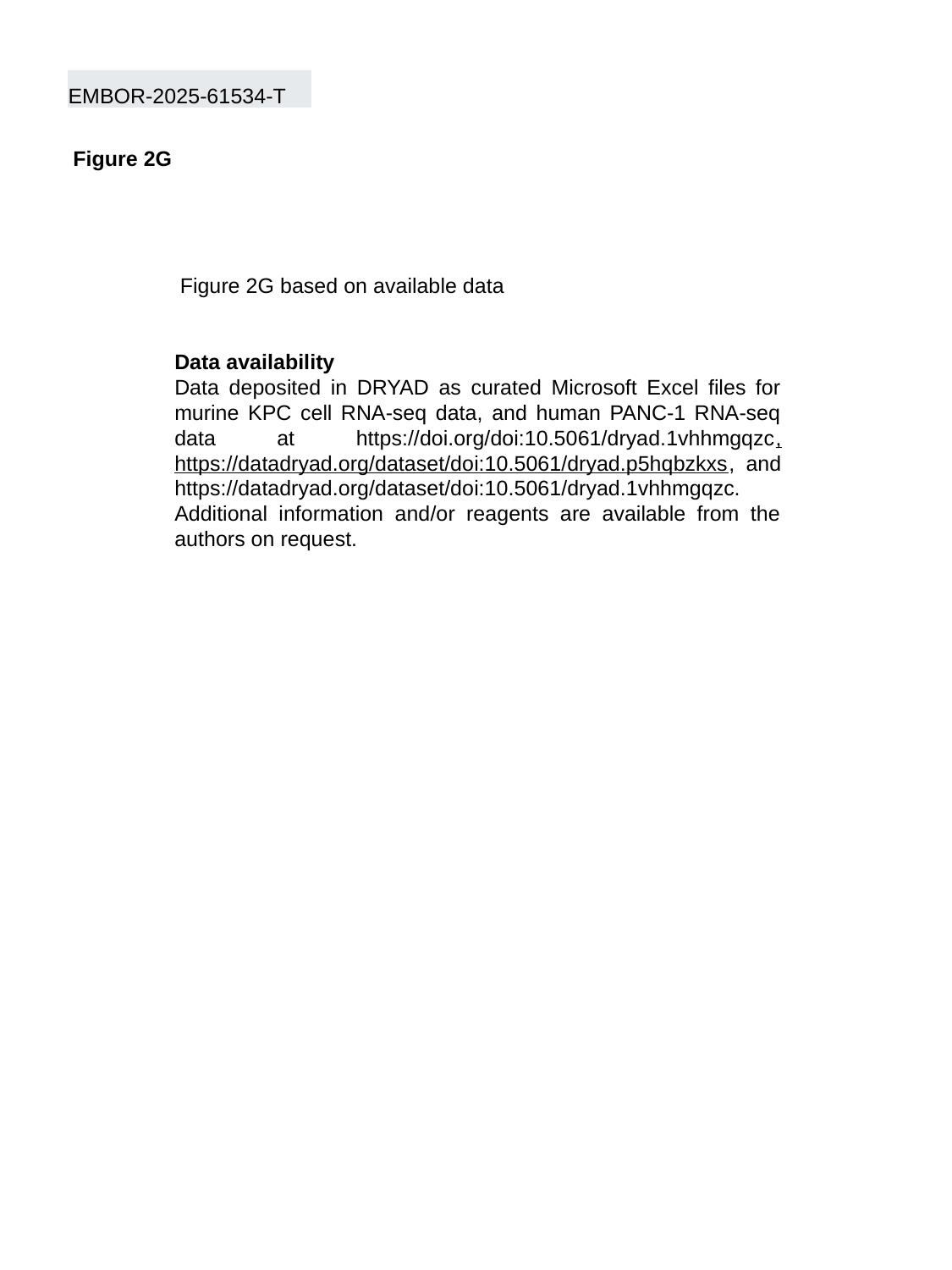

| EMBOR-2025-61534-T |
| --- |
Figure 2G
Figure 2G based on available data
Data availability
Data deposited in DRYAD as curated Microsoft Excel files for murine KPC cell RNA-seq data, and human PANC-1 RNA-seq data at https://doi.org/doi:10.5061/dryad.1vhhmgqzc, https://datadryad.org/dataset/doi:10.5061/dryad.p5hqbzkxs, and https://datadryad.org/dataset/doi:10.5061/dryad.1vhhmgqzc.
Additional information and/or reagents are available from the authors on request.
