# Supplementary material for: STAT3 sustains tumorigenicity following mutant KRAS ablation
Source: EMBO Rep. 2025 Aug 26;26(20):4900–22. doi: 10.1038/s44319-025-00563-w (PMC12549880; doi:10.1038/s44319-025-00563-w)
Supplement: Supplementary file 5 — Source data Fig. 3 [file 44319_2025_563_MOESM5_ESM.zip › Figure 3/Figure 3C/Figure 4C.pptx]

## Slide 1
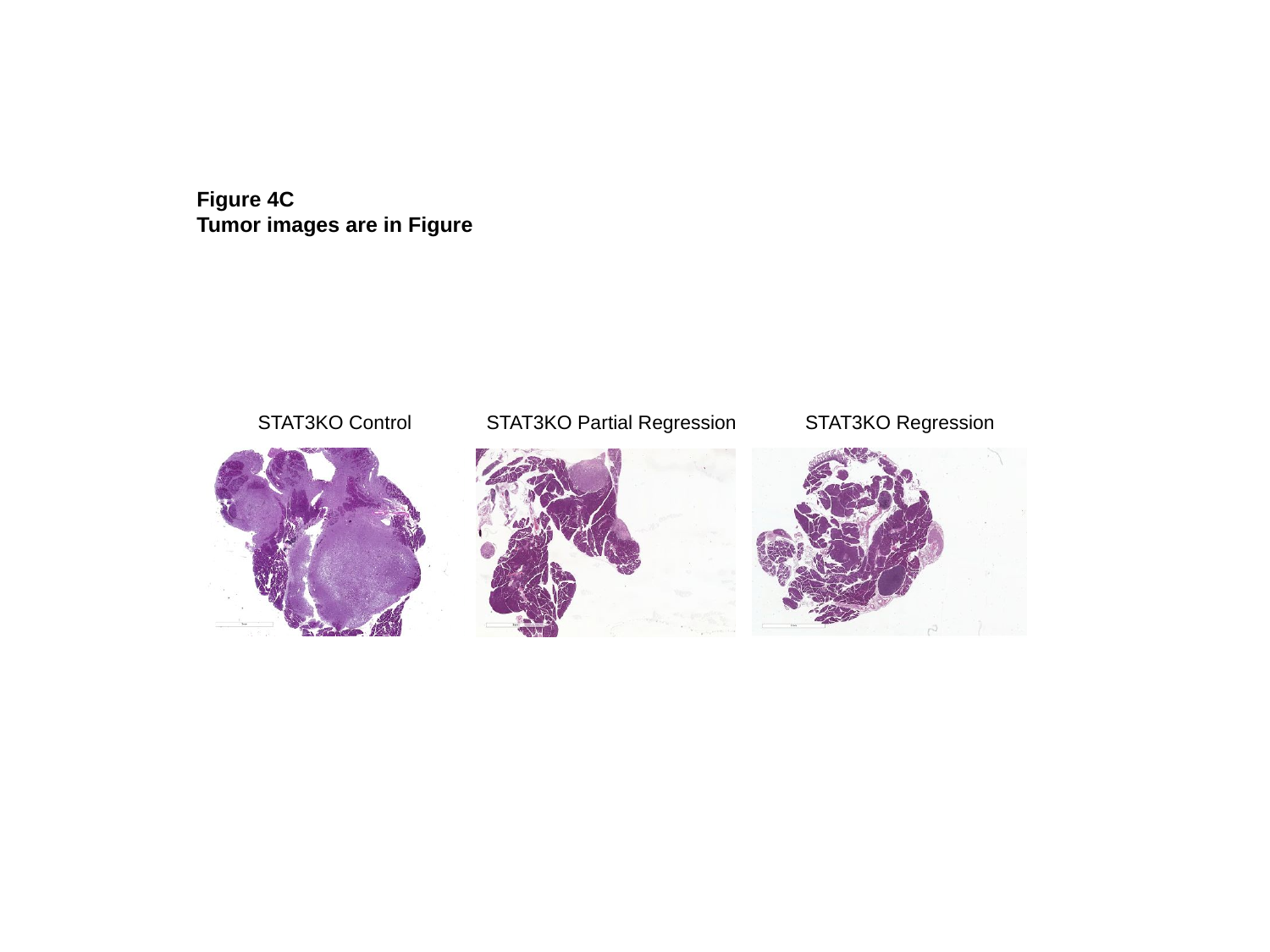

Figure 4C
Tumor images are in Figure
STAT3KO Regression
STAT3KO Partial Regression
STAT3KO Control
