# Supplementary material for: STAT3 sustains tumorigenicity following mutant KRAS ablation
Source: EMBO Rep. 2025 Aug 26;26(20):4900–22. doi: 10.1038/s44319-025-00563-w (PMC12549880; doi:10.1038/s44319-025-00563-w)
Supplement: Supplementary file 6 — Source data Fig. 4 [file 44319_2025_563_MOESM6_ESM.zip › Figure 4/Figure 4D/Figure 4D.pptx]

## Slide 1
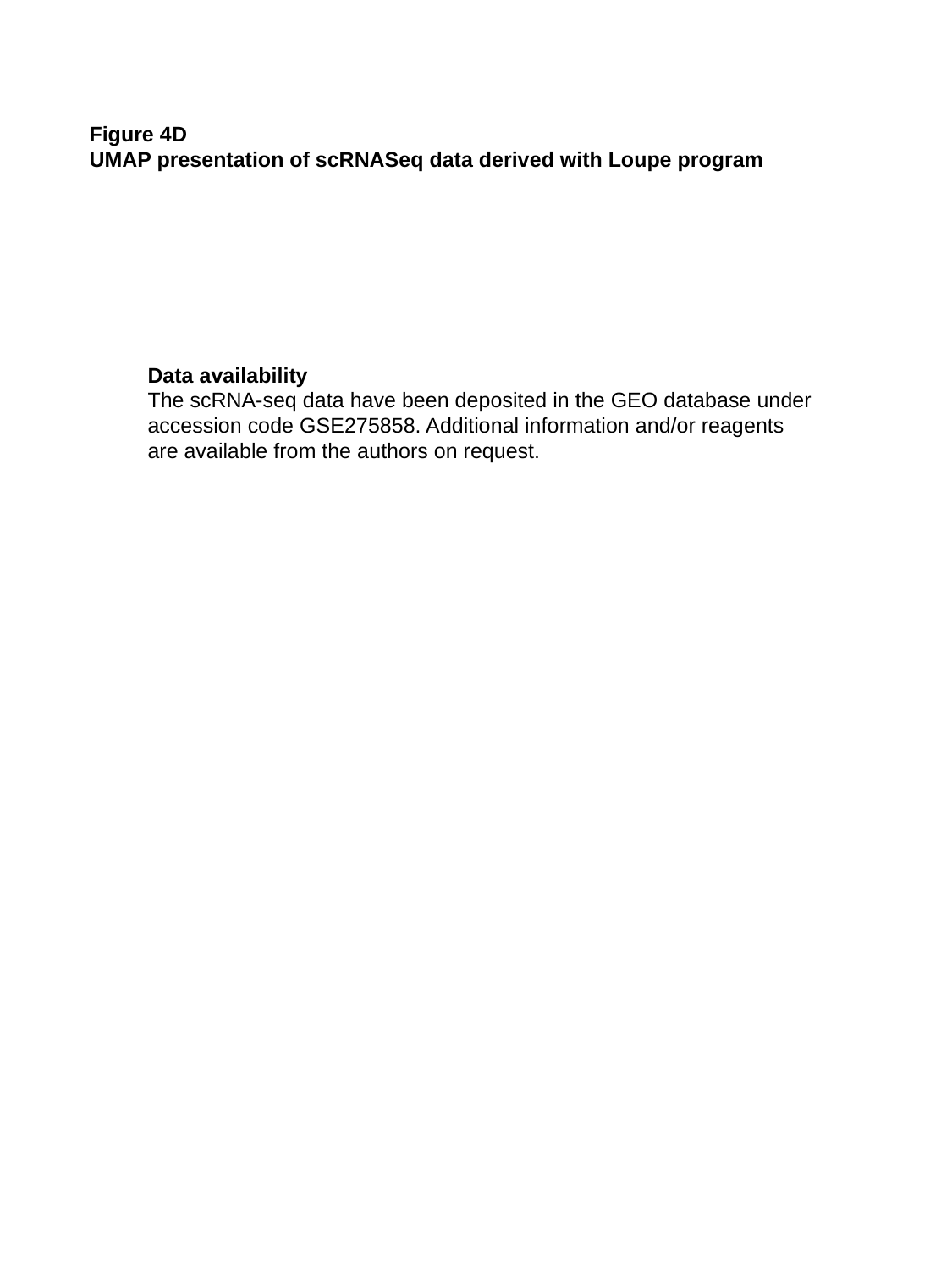

Figure 4D
UMAP presentation of scRNASeq data derived with Loupe program
Data availability
The scRNA-seq data have been deposited in the GEO database under accession code GSE275858. Additional information and/or reagents are available from the authors on request.
