# Supplementary material for: STAT3 sustains tumorigenicity following mutant KRAS ablation
Source: EMBO Rep. 2025 Aug 26;26(20):4900–22. doi: 10.1038/s44319-025-00563-w (PMC12549880; doi:10.1038/s44319-025-00563-w)
Supplement: Supplementary file 6 — Source data Fig. 4 [file 44319_2025_563_MOESM6_ESM.zip › Figure 4/Figure 4G/Figure 4G.pptx]

## Slide 1
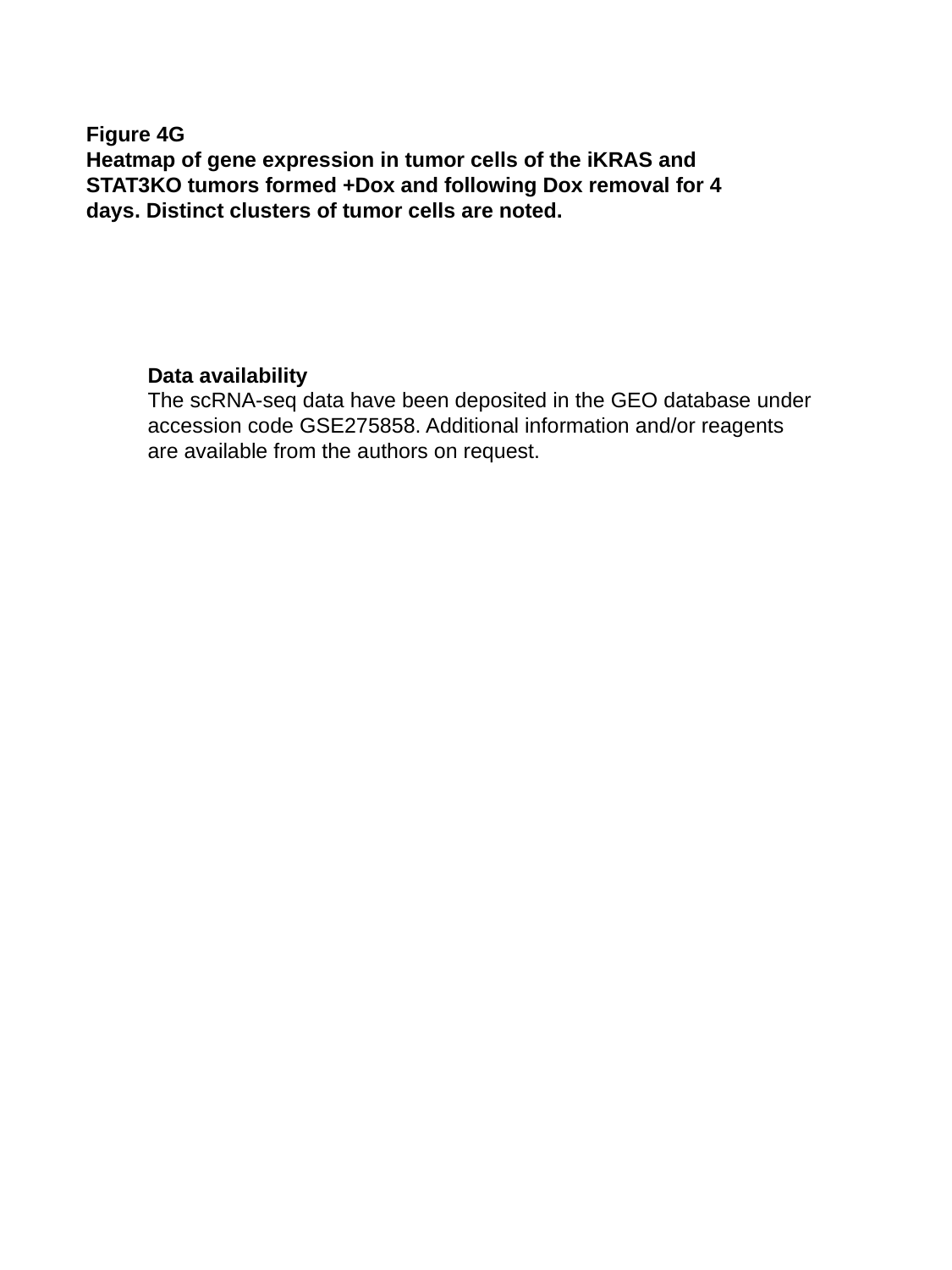

Figure 4G
Heatmap of gene expression in tumor cells of the iKRAS and STAT3KO tumors formed +Dox and following Dox removal for 4 days. Distinct clusters of tumor cells are noted.
Data availability
The scRNA-seq data have been deposited in the GEO database under accession code GSE275858. Additional information and/or reagents are available from the authors on request.
