# Supplementary material for: STAT3 sustains tumorigenicity following mutant KRAS ablation
Source: EMBO Rep. 2025 Aug 26;26(20):4900–22. doi: 10.1038/s44319-025-00563-w (PMC12549880; doi:10.1038/s44319-025-00563-w)
Supplement: Supplementary file 8 — Expanded View Figures [file 44319_2025_563_MOESM8_ESM.pdf]

Expanded View Figures

Figure EV1. STAT3 and KRAS dependency.

(**A**) Gene expression profiling of 168 human pancreatic adenocarcinomas in the TCGA PanCancer Atlas database (http://www.cbioportal.org) corresponding to gene signatures of KRAS dependency (KRAS-type) or reduced KRAS dependency (RSK-type)(Yuan et al, 2018). STAT3 mRNA expression z-score for each tumor sample is displayed on top. (**B**) Scatter plot showing correlation of two independently derived KRAS dependency gene signatures and their references for 150 human pancreatic adenocarcinomas in the TCGA database (http://www.cbioportal.org)(Singh and Settleman, 2009; Yuan et al, 2018). (**C**) Scatter plot showing correlation of STAT3 mRNA expression and RSK-type gene signature (Yuan et al, 2018) in human pancreatic ductal adenocarcinomas from the AUMC (Amsterdam University Medical Centers) database ($n = 80$). (**D**) Proteomic data from human pancreatic adenocarcinomas of the NCI Clinical Proteomic Tumor Analysis Consortium (CPTAC) comparing levels of STAT3 protein in TCGA tumors classified as KRAS-type or RSK-type ($n = 40$). Boxplots show center line as median, box limits as upper and lower quartiles, and whiskers the 1.5 interquartile range. Significance determined using two-tailed *t* test. (**E**) Histo-score (H-score) of the cancer cells in 22 human pancreatic adenocarcinoma tumors from the Stony Brook Medicine Biobank following immunohistochemistry staining for phosphorylated-ERK (P-ERK). (**F**) Representative images of IHC staining of human pancreatic tumors scored in Fig. EV1E with antibodies to phospho-ERK (P-ERK) or STAT3. Tumor cancer cells (Ca) and fibroblast stromal cells (F) are indicated in a tumor. Scale bar 100 mm. (**G**) Representative sequences of major deletions created by CRISPR-mediated editing of mutant KRAS in the human PANC-1 cell line, determined following PCR amplification and cloning. (**H**) Growth in culture of PANC-1 parental cells expressing empty vector or knockout derivatives shown. Strip plots display doubling time for biological replicates ($n = 3$–8) with triplicate technical replicates. Significance was determined using two-tailed test at the 0.05 confidence interval. Plots show median center line and upper and lower quartile lines. (**I**) Representative sequences of the major indels modified by CRISPR-mediated gene editing of STAT3 and mutant KRAS in the KPC cell lines were determined following PCR amplification and cloning. (**J**) Growth in culture of KPC parental cells (PRTL) and derived STAT3 KO, KRAS KO, and DKO4 cells. Cell number during course of two weeks is shown ($n = 3$ biological replicates with triplicate technical replicates). (**K**) Relative tumor development during 60 days by parental KPC cells and derived STAT3 KO, KRAS KO and four DKO KPC cell lines following subcutaneous implantation in nude mice ($n = 8$–12 biological replicates). Data are presented as mean ± SD, two-tailed *t* test. Source data are available online for this figure.

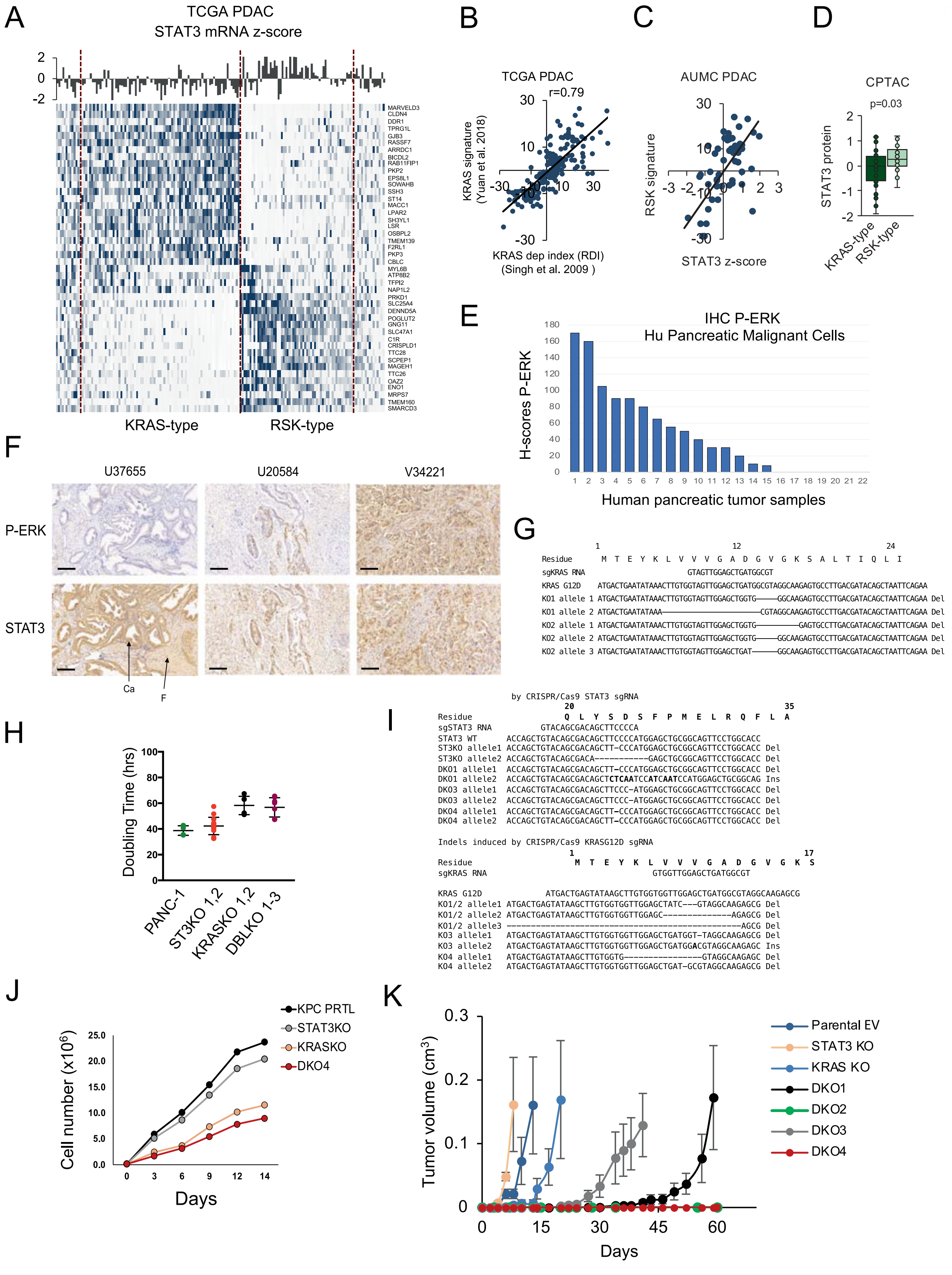

A
TCGA PDAC
STAT3 mRNA z-score
2
0
-2
MARVELD3
CLDN4
DDR1
TPRG1L
GJB3
RASSF7
ARRDC1
BICDL2
RAB11FIP1
PKP2
EPS8L1
SOWAHB
SSH3
ST14
MACC1
LPAR2
SH3YL1
LSR
OSBPL2
TMEM139
F2RL1
PKP3
CBLC
MYL6B
ATP8B2
TFPI2
NAP1L2
PRKD1
SLC25A4
DENND5A
POGLUT2
GNG11
SLC47A1
C1R
CRISPLD1
TTC28
SCPEP1
MAGEH1
TTC26
OAZ2
ENO1
MRPS7
TMEM160
SMARCD3
KRAS-type
RSK-type
B
TCGA PDAC
r=0.79
KRAS signature (Yuan et al. 2018)
KRAS dep index (RDI) (Singh et al. 2009)
C
AUMC PDAC
RSK signature
STAT3 z-score
D
CPTAC
p=0.03
STAT3 protein
KRAS-type
RSK-type
E
IHC P-ERK
Hu Pancreatic Malignant Cells
H-scores P-ERK
Human pancreatic tumor samples
F
U37655
U20584
V34221
P-ERK
STAT3
Ca
F
G
Residue M T E Y K L V V V G A D G V G K S A L T I Q L I
sgKRAS RNA GTAGTTGGAGCTGATGGCGT
KRAS G12D ATGACTGAATATAAACTTGTGGTAGTTGGAGCTGATGGCGTAGGCAAGAGTGCCTTGACGATACAGCTAATTCAGAA
KO1 allele 1 Del
KO1 allele 2 Del
KO2 allele 1 Del
KO2 allele 2 Del
KO2 allele 3 Del
H
Doubling Time (hrs)
PANC-1
ST3KO 1,2
KRASKO 1,2
DBLKO 1-3
I
by CRISPR/Cas9 STAT3 sgRNA
Residue Q L Y S D S F P M E L R Q F L A
sgSTAT3 RNA GTACAGCGACAGCTTCCCCA
STAT3 WT ACCAGCTGTACAGCGACAGCTTCCCCATGGAGCTGCGGCAGTTCCTGGCACC
ST3KO allele1 Del
ST3KO allele2 Del
DKO1 allele1 Del
DKO1 allele2 Ins
DKO3 allele1 Del
DKO3 allele2 Del
DKO4 allele1 Del
DKO4 allele2 Del
Indels induced by CRISPR/Cas9 KRASG12D sgRNA
Residue M T E Y K L V V V G A D G V G K S
sgKRAS RNA GTGGTTGGAGCTGATGGCGT
KRAS G12D ATGACTGAGTATAAGCTTGTGGTGGTTGGAGCTGATGGCGTAGGCAAGAGCG
KO1/2 allele1 Del
KO1/2 allele2 Del
KO1/2 allele3 Del
KO3 allele1 Del
KO3 allele2 Ins
KO4 allele1 Del
KO4 allele2 Del
J
KPC PRTL
STAT3KO
KRASKO
DKO4
Cell number (x10^6)
Days
K
Parental EV
STAT3 KO
KRAS KO
DKO1
DKO2
DKO3
DKO4
Tumor volume (cm^3)
Days

 © The Author(s)

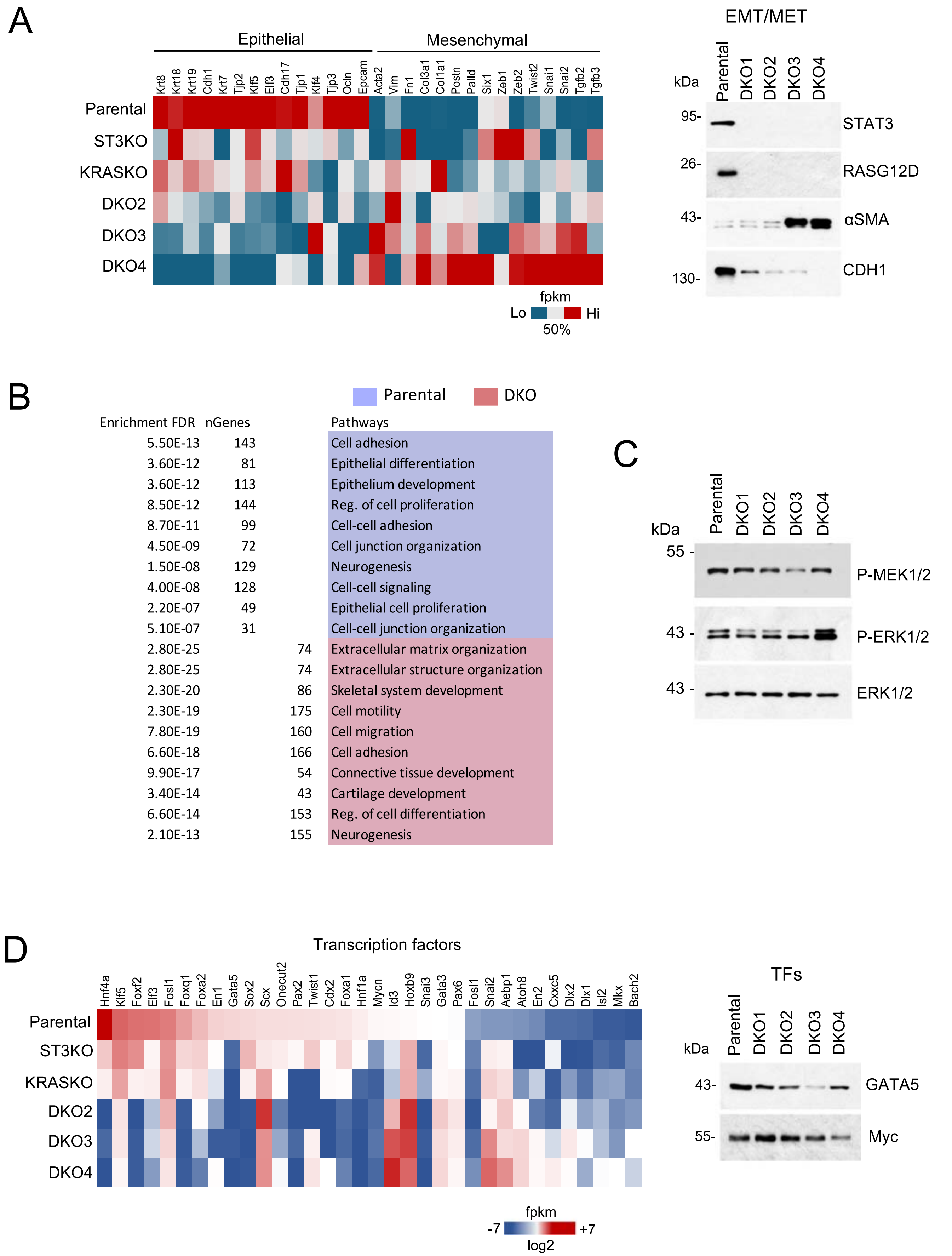
A
Epithelial
Mesenchymal
Krt8 Krt18 Krt19 Cdh1 Krt7 Tjp2 Klf5 Elf3 Cdh17 Tjp1 Klf4 Tjp3 Ocln Epcam
Acta2 Vim Fn1 Col3a1 Col1a1 Postn Palld Six1 Zeb1 Zeb2 Twist2 Snai1 Snai2 Tgfb2 Tgfb3
Parental
ST3KO
KRASKO
DKO2
DKO3
DKO4
fpkm
Lo Hi
50%
EMT/MET
kDa
Parental DKO1 DKO2 DKO3 DKO4
95- STAT3
26- RASG12D
43- αSMA
130- CDH1
B
Parental DKO
Enrichment FDR nGenes Pathways
5.50E-13 143 Cell adhesion
3.60E-12 81 Epithelial differentiation
3.60E-12 113 Epithelium development
8.50E-12 144 Reg. of cell proliferation
8.70E-11 99 Cell-cell adhesion
4.50E-09 72 Cell junction organization
1.50E-08 129 Neurogenesis
4.00E-08 128 Cell-cell signaling
2.20E-07 49 Epithelial cell proliferation
5.10E-07 31 Cell-cell junction organization
2.80E-25 74 Extracellular matrix organization
2.80E-25 74 Extracellular structure organization
2.30E-20 86 Skeletal system development
2.30E-19 175 Cell motility
7.80E-19 160 Cell migration
6.60E-18 166 Cell adhesion
9.90E-17 54 Connective tissue development
3.40E-14 43 Cartilage development
6.60E-14 153 Reg. of cell differentiation
2.10E-13 155 Neurogenesis
C
kDa
Parental DKO1 DKO2 DKO3 DKO4
55 - P-MEK1/2
43 - P-ERK1/2
43 - ERK1/2
D
Transcription factors
Hnf4a Klf5 Foxf2 Elf3 Fosl1 Foxq1 Foxa2 En1 Gata5 Sox2 Scx Onecut2 Pax2 Twist1 Cdx2 Foxa1 Hnf1a Mycn Id3 Hoxb9 Snai3 Gata3 Pax6 Fosl1 Snai2 Aebp1 Atoh8 En2 Cxxc5 Dlx2 Dlx1 Isl2 Mkx Bach2
Parental
ST3KO
KRASKO
DKO2
DKO3
DKO4
fpkm
-7 +7
log2
TFs
kDa
Parental DKO1 DKO2 DKO3 DKO4
43- GATA5
55- Myc

◀ **Figure EV2. Transcriptional reprogramming following loss of KRAS and STAT3.**

(**A**) Left) Heatmap of gene signatures for epithelial and mesenchymal identity derived from RNA-seq data of KPC parental, STAT3 KO cells, KRAS KO cells, and three of the DKO clones. Differences are compared for an individual gene across multiple cell types due to variance in gene representation. Highest (Hi) to lowest (Lo) counts (fpkm) and 50 percentile are shown for each gene. Right) Western blots of KPC parental cells and four DKO cell lines confirming RNA-seq data for expression of epithelial E-cadherin (CDH1) and mesenchymal smooth muscle actin (aSMA/ACTA2). (**B**) Gene ontology (GO) classification of top biological processes that are upregulated in KPC parental cells (blue) or DKO4 (red) cells. (**C**) Western blot of KPC parental cells and four DKO derived clones for phosphorylation activity of MEK and ERK1/2. Samples were run on the same gel as Fig. 2E with ERK1/2 control. (**D**) Left) Heatmaps of differential gene expression from RNA Seq analyses corresponding to a set of transcription factors in parental KPC cells, STAT3 KO cells, KRAS KO and three DKO derived clones. Right) Western blots of KPC parental cells and four DKO cell lines for GATA5, and MYC. Samples were run on the same gel as Fig. 2E. Source data are available online for this figure.

 © The Author(s)

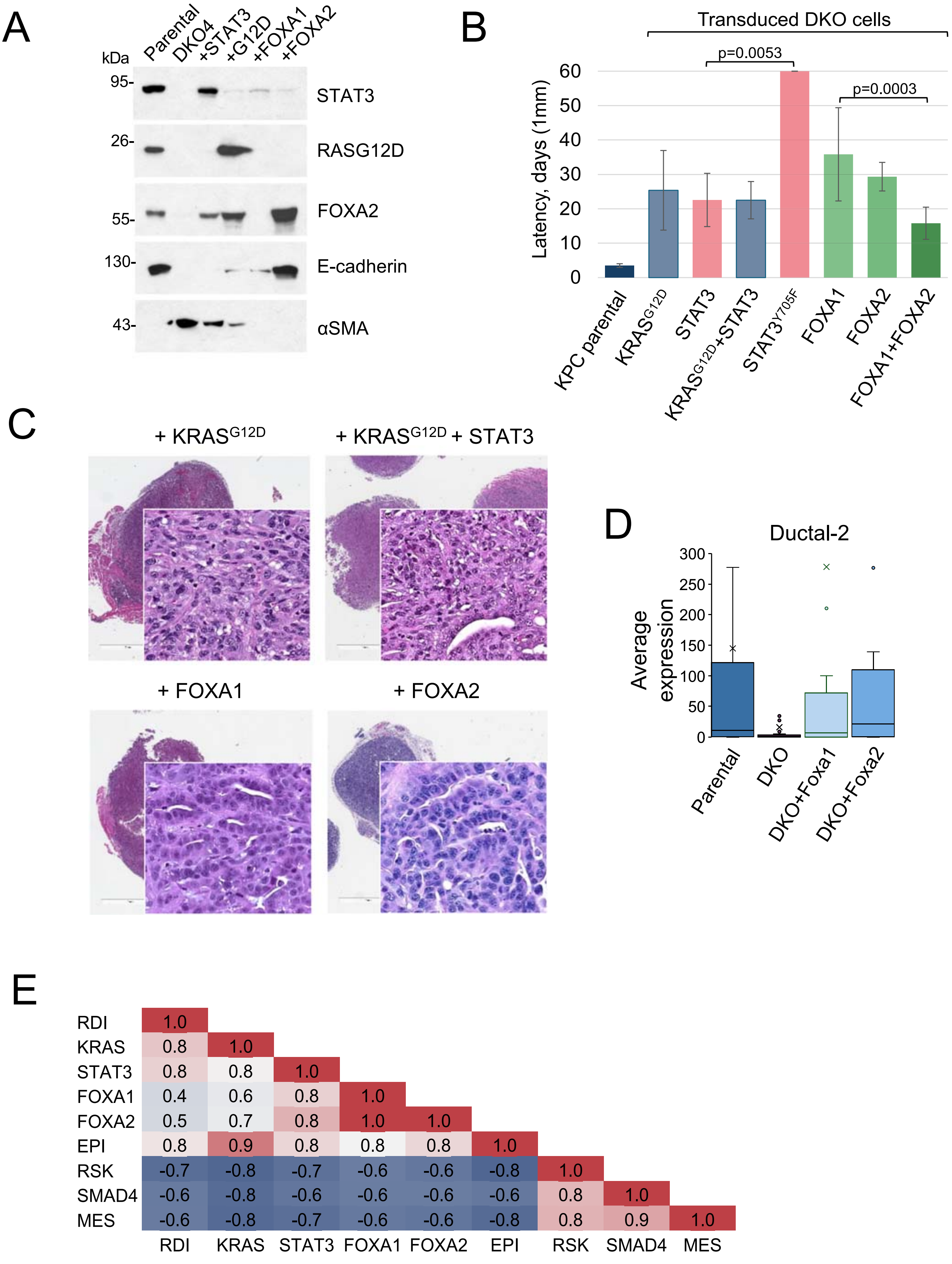
A
kDa
Parental
DKO4
+STAT3
+G12D
+FOXA1
+FOXA2
95-
26-
55-
130-
43-
STAT3
RASG12D
FOXA2
E-cadherin
αSMA
B
Transduced DKO cells
p=0.0053
p=0.0003
Latency, days (1mm)
0
10
20
30
40
50
60
KPC parental
KRASG12D
STAT3
KRASG12D+STAT3
STAT3Y705F
FOXA1
FOXA2
FOXA1+FOXA2
C
+ KRASG12D
+ KRASG12D + STAT3
+ FOXA1
+ FOXA2
D
Ductal-2
Average expression
0
50
100
150
200
250
300
Parental
DKO
DKO+Foxa1
DKO+Foxa2
E
RDI 1.0
KRAS 0.8 1.0
STAT3 0.8 0.8 1.0
FOXA1 0.4 0.6 0.8 1.0
FOXA2 0.5 0.7 0.8 1.0 1.0
EPI 0.8 0.9 0.8 0.8 0.8 1.0
RSK -0.7 -0.8 -0.7 -0.6 -0.6 -0.8 1.0
SMAD4 -0.6 -0.8 -0.6 -0.6 -0.6 -0.6 0.8 1.0
MES -0.6 -0.8 -0.7 -0.6 -0.6 -0.8 0.8 0.9 1.0
RDI KRAS STAT3 FOXA1 FOXA2 EPI RSK SMAD4 MES

◀ **Figure EV3. Transcriptional reprogramming following loss of KRAS and STAT3.**

(**A**) Representative Western blot of parental KPC cells, DKO cells, and DKO cells restored for expression of the designated gene by lentiviral transduction. (**B**) Restoration of subcutaneous tumorigenicity in nude mice of DKO4 cells quantified by tumor latency in days (~1 mm size tumor) following transduction with *Kras*^{G12D}, *Stat3, Stat3*^{Y705F}, *Foxa1, Foxa2* or the indicated combinations compared with parental KPC cells ($n = 8$–12). No tumors were formed by DKO4 cells. Significance was determined using two-tailed test at the 0.05 confidence interval. (**C**) Representative H&E histology images of tumors formed following transduction of DKO4 cells with genes noted. (**D**) Average expression of the top ductal-2 type PDAC genes determined by RNA-seq of KPC parental cells, DKO cells, or DKO cells transduced with *Foxa1* or *Foxa2* ($n = 2$–4)(Peng et al, 2019). Significance was determined using two-tailed test at the 0.05 confidence interval. (**E**) Pearson's coefficient analyses aligning gene expression signatures of reconstituted KPC cells as noted with PDAC TCGA tumor samples ($n = 168$, stage I/II tumors) according to RAS dependency (RDI), molecular subtype (i.e., KRAS-type vs RSK-type), and epithelial (EPI) or mesenchymal (MES) status. Source data are available online for this figure.

 © The Author(s)

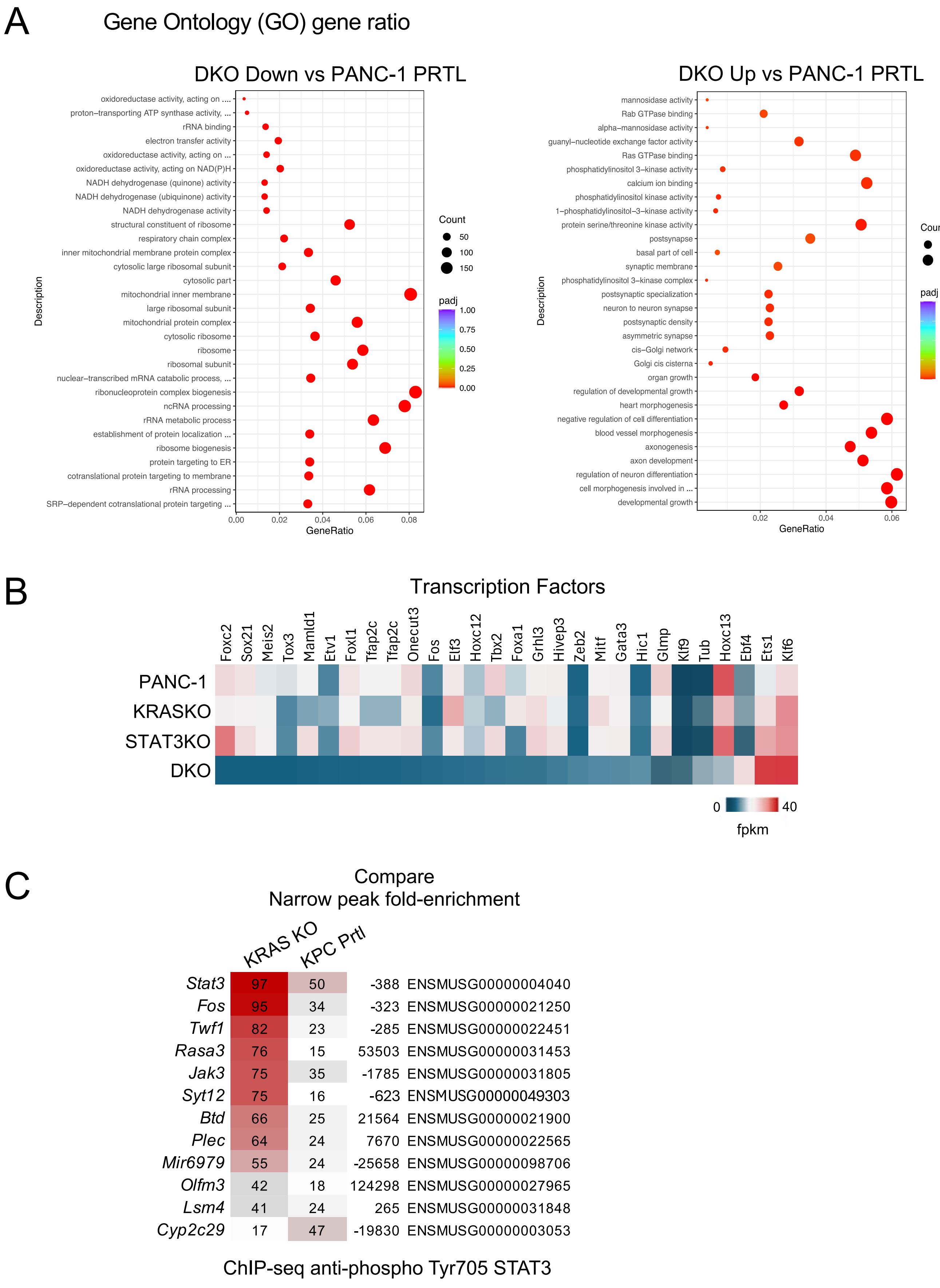
A
Gene Ontology (GO) gene ratio
DKO Down vs PANC-1 PRTL
oxidoreductase activity, acting on ....
proton−transporting ATP synthase activity, ...
rRNA binding
electron transfer activity
oxidoreductase activity, acting on ...
oxidoreductase activity, acting on NAD(P)H
NADH dehydrogenase (quinone) activity
NADH dehydrogenase (ubiquinone) activity
NADH dehydrogenase activity
structural constituent of ribosome
respiratory chain complex
inner mitochondrial membrane protein complex
cytosolic large ribosomal subunit
cytosolic part
mitochondrial inner membrane
large ribosomal subunit
mitochondrial protein complex
cytosolic ribosome
ribosome
ribosomal subunit
nuclear−transcribed mRNA catabolic process, ...
ribonucleoprotein complex biogenesis
ncRNA processing
rRNA metabolic process
establishment of protein localization ...
ribosome biogenesis
protein targeting to ER
cotranslational protein targeting to membrane
rRNA processing
SRP−dependent cotranslational protein targeting ...
Description
GeneRatio
0.00
0.02
0.04
0.06
0.08
Count
50
100
150
padj
1.00
0.75
0.50
0.25
0.00
DKO Up vs PANC-1 PRTL
mannosidase activity
Rab GTPase binding
alpha−mannosidase activity
guanyl−nucleotide exchange factor activity
Ras GTPase binding
phosphatidylinositol 3−kinase activity
calcium ion binding
phosphatidylinositol kinase activity
1−phosphatidylinositol−3−kinase activity
protein serine/threonine kinase activity
postsynapse
basal part of cell
synaptic membrane
phosphatidylinositol 3−kinase complex
postsynaptic specialization
neuron to neuron synapse
postsynaptic density
asymmetric synapse
cis−Golgi network
Golgi cis cisterna
organ growth
regulation of developmental growth
heart morphogenesis
negative regulation of cell differentiation
blood vessel morphogenesis
axonogenesis
axon development
regulation of neuron differentiation
cell morphogenesis involved in ...
developmental growth
Description
GeneRatio
0.02
0.04
0.06
Count
50
100
padj
1.00
0.75
0.50
0.25
0.00
B
Transcription Factors
Foxc2 Sox21 Meis2 Tox3 Mamld1 Etv1 Foxl1 Tfap2c Tfap2c Onecut3 Fos Elf3 Hoxc12 Tbx2 Foxa1 Grhl3 Hivep3 Zeb2 Mitf Gata3 Hic1 Glmp Klf9 Tub Hoxc13 Ebf4 Ets1 Klf6
PANC-1
KRASKO
STAT3KO
DKO
0
40
fpkm
C
Compare
Narrow peak fold-enrichment
KRAS KO
KPC Prtl
Stat3 97 50 -388 ENSMUSG00000004040
Fos 95 34 -323 ENSMUSG00000021250
Twf1 82 23 -285 ENSMUSG00000022451
Rasa3 76 15 53503 ENSMUSG00000031453
Jak3 75 35 -1785 ENSMUSG00000031805
Syt12 75 16 -623 ENSMUSG00000049303
Btd 66 25 21564 ENSMUSG00000021900
Plec 64 24 7670 ENSMUSG00000022565
Mir6979 55 24 -25658 ENSMUSG00000098706
Olfm3 42 18 124298 ENSMUSG00000027965
Lsm4 41 24 265 ENSMUSG00000031848
Cyp2c29 17 47 -19830 ENSMUSG00000003053
ChIP-seq anti-phospho Tyr705 STAT3

Figure EV4. Transcriptional reprogramming following loss of KRAS and STAT3.

(**A**) Gene ontology (GO) comparison of biological processes expressed in human PANC-1 DKO cells versus parental PANC-1 cells derived from RNASeq analyses. Comparisons shown as dot matrices. (**B**) Heatmaps of differential gene expression from RNA Seq analyses corresponding to a set of transcription factors in parental human PANC-1 EV cells or derived KRAS KO, STAT3 KO, or DKO cells. (**C**) Differential enrichment of genes in KRASKO and KPC parental cells identified by ChIP with antibodies to tyrosine 705 phosphorylated STAT3. Comparisons are shown as fold enrichment in narrow peaks for genes relative to control antibody or DKO cells (count per 1 M reads IP/input). Gene identity and location of the transcriptional start sites are indicated.

 © The Author(s)

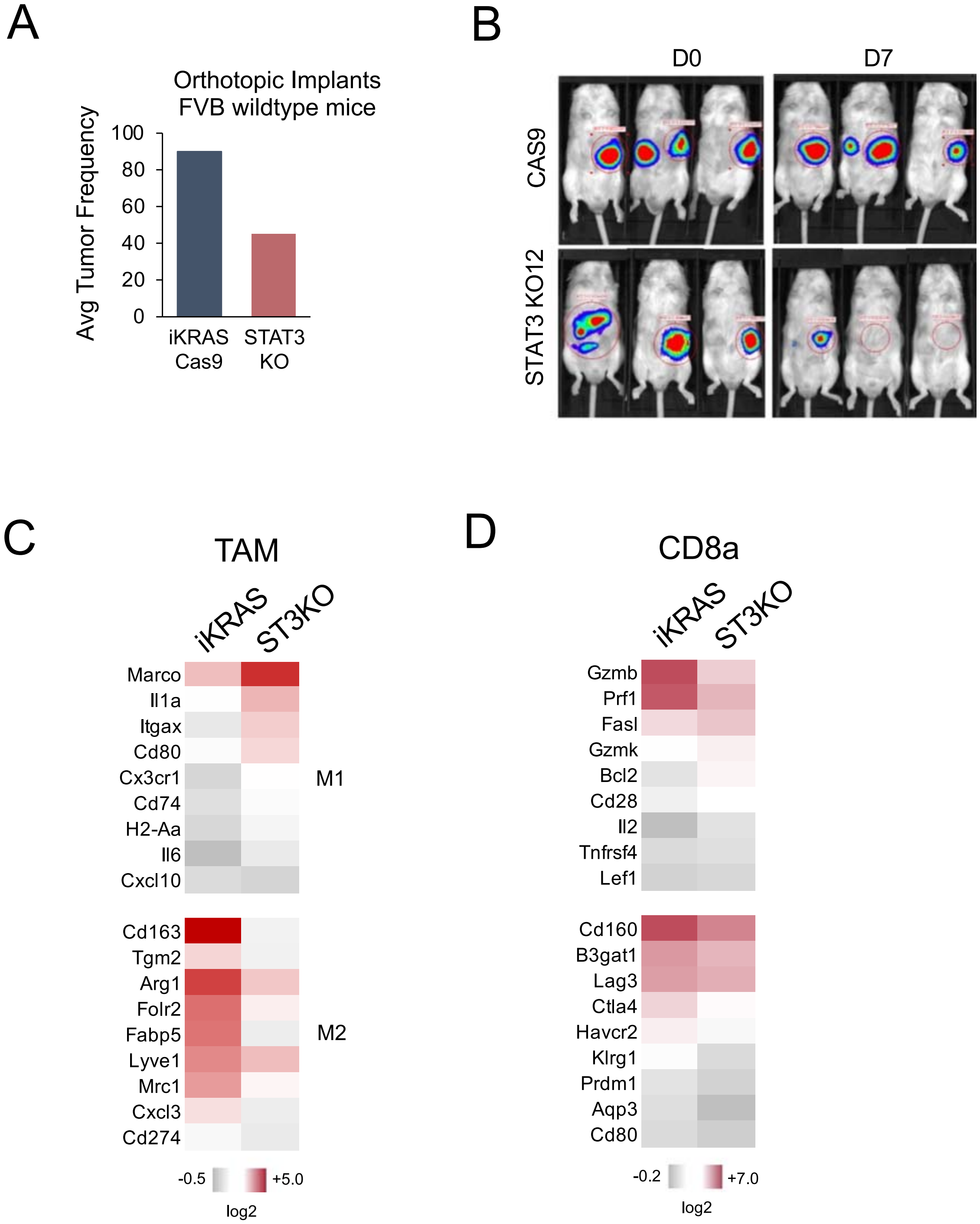

Figure EV5. STAT3 depletion in an inducible mutant KRAS PDAC model.

(**A**) Average frequency of tumor formation in FVB wild-type mice treated with doxycycline of parental iKRAS cells expressing empty vector (EV) or three independent CRISPR-edited STAT3 KO iKRAS clones. (**B**) Representative in vivo bioluminescence imaging of tumors formed by orthotopic implants of iKRAS parental cells expressing empty vector (Cas9) or derived STAT3 KO12 cells that were transduced to express luciferase. Mice were administered doxycycline and tumors were evaluated on day 0 (D0) or seven days after withdrawal of doxycycline (D7). (**C**) Comparative heatmaps of scRNA-seq individual datasets for a subset of genes expressed in the tumor associated macrophages (TAMs) in tumors formed by iKRAS control cells or STAT3KO derived cells in FVB mice treated with doxycycline based on captured viable cell populations and proportions. (**D**) Comparative heatmaps of scRNA-seq individual datasets for a subset of genes expressed in CD8a+ T cells (CD8a expression log2 fold greater than CD4 expression) in tumors formed by iKRAS control cells or STAT3KO derived cells in FVB mice treated with doxycycline based on captured viable cell populations and proportions. Source data are available online for this figure.

© The Author(s)
